# Selective binding of a toxin and phosphatidylinositides to a mammalian potassium channel

**DOI:** 10.1038/s41467-019-09333-4

**Published:** 2019-03-22

**Authors:** Yang Liu, Catherine E. LoCaste, Wen Liu, Michael L. Poltash, David H. Russell, Arthur Laganowsky

**Affiliations:** 1 Institute of Biosciences and Technology, Texas A&M Health Science Center, Houston, TX 77030 USA; 2 Department of Chemistry, Texas A&M University, College Station, TX 77842 USA

## Abstract

G-protein-gated inward rectifying potassium channels (GIRKs) require G_βγ_ subunits and phosphorylated phosphatidylinositides (PIPs) for gating. Although studies have provided insight into these interactions, the mechanism of how these events are modulated by G_βγ_ and the binding affinity between PIPs and GIRKs remains poorly understood. Here, native ion mobility mass spectrometry is employed to directly monitor small molecule binding events to mouse GIRK2. GIRK2 binds the toxin tertiapin Q and PIPs selectively and with significantly higher affinity than other phospholipids. A mutation in GIRK2 that causes a rotation in the cytoplasmic domain, similarly to G_βγ_-binding to the wild-type channel, revealed differences in the selectivity towards PIPs. More specifically, PIP isoforms known to weakly activate GIRKs have decreased binding affinity. Taken together, our results reveal selective small molecule binding and uncover a mechanism by which rotation of the cytoplasmic domain can modulate GIRK•PIP interactions.

## Abstract (web summary)

G-protein-gated inward rectifying potassium channels (GIRKs) require G_βγ_ subunits and phosphorylated phosphatidylinositides (PIPs) for gating. Here authors use native ion mobility mass spectrometry to monitor small molecule binding events to GIRK2 and shed light on the selectivity of GIRK2 towards PIPs.

## Introduction

Inward rectifying potassium (IRK) channels are a family of membrane protein complexes that transport potassium ions across biological membranes into cells whereas outward flux is blocked by intracellular polyamines and magnesium^1,2^. These channels are involved in numerous physiological processes, such as neuronal signaling, heart rate regulation, and potassium homeostasis^2–4^. Mutations in IRK channels underlie a number of diseases including Andersen syndrome, Bartter syndrome, and neonatal diabetes^5–7^. Required for activation of IRK and a number of other channels is the signaling lipid phosphatidylinositol 4,5-bisphosphate (PI(4,5)P_2_), which represents a minor component of the cytoplasmic leaflet^8–16^. IRK channels are also regulated by a number of other molecules including anionic lipids, sodium, ethanol, and toxins^2,17–24^.

Atomic structures have revealed detailed interactions of PI(4,5)P_2_ with dioctanoyl (8:0–8:0) acyl chains binding to chicken IRK2 (Kir2.2) and mouse G-protein (G) gated IRK2 (GIRK2, Kir3.2)^12,25,26^. These structures reveal a similar fold with one PI(4,5)P_2_ bound per subunit at the interface between the transmembrane domain and cytoplasmic domain (CTD). The negatively charged, phosphate-rich head group of PI(4,5)P_2_ promotes coordination with lysine residues and backbone amides on GIRK2^25^. PI(4,5)P_2_ binding to GIRK2 induces a slight rotation of the inner helix but insufficient to open the channel. An open, gated conformation is only achieved as a result of a four degree rotation of the CTD upon G_βγ_ binding between adjacent CTD subunits. The R201A mutation of GIRK2 (GIRK2^R201A^) does not require G_βγ_ binding to gate and the structure in complex with PI(4,5)P_2_ is in a gated conformation. However, there is an exaggerated rotation of the CTD compared to the gated wild-type channel. GIRK2^R201A^ has been proposed to be a mimic of the G_βγ_-protein activated state^25^, and provides an opportunity to study the effect of the CTD orientation on PIP binding.

In addition to PI(4,5)P_2_, electrophysiology studies have revealed the other six PIP isoforms can gate IRK subfamilies to various degrees^10,16,19,27,28^. For example, IRK1 (Kir2.1) is activated primarily by PI(4,5)P_2_ but the channel appears to bind other PIPs with similar affinity^10,28^. The hetero-tetrameric GIRK1/GIRK4 channel, which is in the same subfamily as GIRK2, can be activated with similar efficacy by PI(4,5)P_2_, PI(3,4)P_2_, PI(3,5)P_2_, and PI(3,4,5)P_3_^16^. ATP-sensitive potassium channels (K_ATP_) are IRKs that are promiscuously activated by PIPs^10^. Moreover, GIRK1/GIRK4 has a strong dependence on the acyl chain chemistry of the PIP with the greatest activation for 18:0–20:4 tails^16^, which is the most abundant form in mammalian cells^29^. In contrast, IRK1 does not display a preference toward the acyl chains of PIPs^16,28^. Although functional assays report an overall pharmacological shift and not on the stoichiometry of bound PIPs, these studies demonstrate that IRKs are distinctly activated by PIP isoforms. In addition, anionic lipids, such as phosphatidylglycerol, not only are required for gating of Kir2 channels but also increases sensitivity of Kir2 toward PIPs by 10–100-fold^19,30^. With these findings in mind, there is an emerging necessity for novel approaches to interrogate individual PIP binding events to these tetrameric ion channels in order to gain chemical insight into the impact of PIP isoforms and acyl chain chemistry on binding.

Native mass spectrometry (MS) is emerging as a powerful biophysical technique, especially for investigating membrane proteins and their interactions with small molecules, such as lipids^31–34^. Unlike other biophysical techniques, native MS can interrogate individual ligand-binding events to protein complexes while preserving noncovalent interactions in the mass spectrometer^32,35–38^. For example, native MS coupled with a temperature controlled source has been used to obtain thermodynamics for membrane protein interactions with lipids and protein^33,39^. Notably, thermodynamic parameters for protein–ligand and protein–protein interactions determined using other biophysical techniques, such as isothermal titration calorimetry (ITC) and surface plasmon resonance (SPR), are in agreement with those obtained using native MS^33,39,40^. Although an improperly tuned instrument could adversely affect the fraction of lipid bound to the protein^38^, prior native MS studies^33,39,40^ establish that a properly tuned instrument can measure equilibrium binding constants and thermodynamic parameters comparable to those measured in the solution phase. Moreover, MS has also revealed that specific protein–lipid interactions can stabilize protein complexes^32,41^ and allosterically modulate other interactions with protein^39^, lipids^42^, and drugs^31,43^.

Although native MS has provided insight into a handful of membrane protein complexes, the rate-limiting step to obtaining a mass spectrum—a prerequisite for detailed biophysical studies—is often frustrated by sample preparation and quality^44^. These challenges in general are exacerbated for eukaryotic membrane proteins, such as for G-protein coupled receptors, requiring considerable effort to optimize their purification^45^. Here, we optimize the purification of the mammalian GIRK2 channel for native MS studies. We then use native MS to study the binding of small molecules and PIP isoforms including different acyl chains to GIRK2 and the R201A mutant of GIRK2 that displays a rotation of the CTD similar to that when in complex with PIP and G_βγ_^25^.

## Results

### Optimization of GIRK2 for native MS

**Fig. 1:**
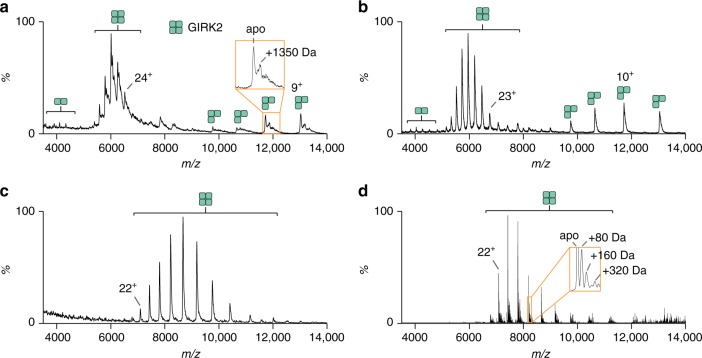
Optimization of mouse GIRK2 for native mass spectrometry studies. **a** Initial preparation of GIRK2 in dodecylmaltoside (DDM) following established protocols for structural studies^25^. The mass spectrum was recorded using maximum energy settings to reveal 1350 Da adducts bound to GIRK2. **b** Mass spectrum of GIRK2 in DDM after DHPC treatment, which reveals a well-resolved mass spectrum with the lipid adducts removed. DHPC treated GIRK2 in the charge-reducing detergent, C_10_E_5_ acquired under minimal energy regimes on a **c** Waters Synapt G1 and **d** Thermo Exactive plus EMR modified with a rear-entry source^49^. The mass spectra resolves small molecule adducts bound to the tetrameric complex

Following established methods used for crystallographic studies of GIRK2^25,26^, we first recorded a mass spectrum of GIRK2 solubilized in dodecylmaltoside (DDM) detergent micelles. High-energy instrument settings were necessary to obtain an interpretable mass spectrum on a Waters Synapt G1 instrument (Fig. 1a and Supplementary Fig. 1). The dominant mass spectral peaks centered around 6000 m/z correspond to the tetrameric channel. Significant adducts remain bound, even under this high-energy regime, corresponding to a host of small molecule contaminants, which we suspect are co-purified lipids. We then used a detergent screening approach^44^ to identify conditions that remove the co-purified contaminants from the complex (Supplementary Fig. 1). From this exhaustive screen of detergents performed at different steps in the purification process, including mixtures of detergents, no conditions were found to effectively remove all of the bound contaminants while maintaining protein solubility. We then explored lipids with detergent-like properties and found the short-chain phospholipid DHPC (1,2-diheptanoyl-sn-glycero-3-phosphocholine) to be the most effective at removing adducts while minimizing protein aggregation. The mass spectrum of GIRK2 in DDM after DHPC treatment had no lipid adducts even under moderate instrument settings (Fig. 1b). Although the mass spectrum is resolved, it is difficult to preserve native-like structure using DDM, a noncharge-reducing detergent, as evident by broad arrival time distributions and dissociation of the complex (Fig. 1b and Supplementary Fig. 2A). Similar observations have been made for other membrane proteins^32,46^. Therefore, we exchanged GIRK2 into buffer containing the charge-reducing detergent C_10_E_5_ (decylpentaglycol) (Fig. 1c). The mass spectrum of this sample is well-resolved and the ion mobility (IM) measurements, which report on the rotationally averaged collision cross-section, indicate compact, native-like arrival times for half of the charge states under the optimized instrument settings (Fig. 1c and Supplementary Fig. 2B). The measured mass agrees with the calculated mass (Supplementary Table 1). The C_10_E_5_ detergent micelle requires more energy to release from the protein than the commonly used C_8_E_4_ detergent, providing an explanation for the partial activation of the higher charge states^46^. Notably, the sample following established methods in DDM under the same instrument conditions used for the optimized sample in C_10_E_5_ was largely a hump with some poorly resolved mass spectral peaks observed (Supplementary Fig. 3A). Moreover, the C_10_E_5_ detergent reduces enough charge such that no dissociation of the complex is observed even at the highest energy regime (Supplementary Fig. 3B), which is in accord with other charge-reduced membrane protein studies^32,46,47^.

### Identification of phosphorylation sites of GIRK2

We also acquired data on a higher mass resolution Orbitrap mass spectrometer^48^ equipped with a custom reverse-entry ion source (REIS-Orbitrap)^49,50^ that yielded sharp mass spectral peaks with addition of mass as small as 80 Da resolved (Fig. 1d). As the 80 Da adduct with full width half maximum of 17 Da could be a covalent modification, we prepared a denatured sample of GIRK2 following a method recently reported by Campuzano and co-workers^51^. The mass spectrum of the denatured sample recorded on the Orbitrap mass spectrometer revealed a predominant denatured mass of 38,939.65 Da (Supplementary Fig. 4), which is within 2.20 Da of the calculated mass of GIRK2 with the initiating methionine removed. Removal of the first residue by methionine aminopeptidase is not an uncommon post-translational modification^52,53^. Moreover, a denatured mass consistent with one phosphorylation of GIRK2 was also measured and accounts for a small fraction of the total signal. This percentage of phosphorylation is consistent with the decrease in intensity of one and multiple phosphorylation events within the tetrameric complex. We then performed a tryptic peptide analysis to confirm phosphorylation and identify site(s) of the covalent modification. We obtained a sequence coverage of 53% and a number of peptides having only a single phosphorylation were identified (Supplementary Fig. 5A and Supplementary Table 2). Of those, four phosphorylated residues mapped to surface exposed regions of the cytoplasmic domain near the C-terminus (Supplementary Figs. 5B and 6). Two serine residues that are part of the linker region between GIRK2 and the TEV protease cleavage site of the expression construct were unexpectedly both found to be singly phosphorylated.

### Gas-phase stability of GIRK2 bound to TertiapinQ

**Fig. 2:**
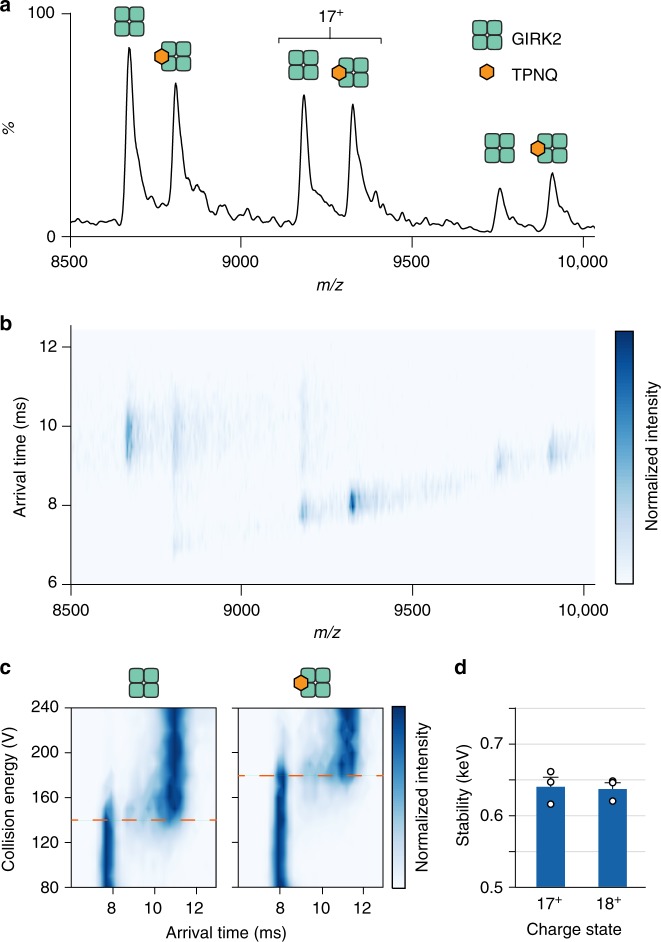
Honey bee toxin, Tertiapin Q (TPNQ) binding and stabilization of GIRK2. **a** Mixture of GIRK2 and six molar equivalents of TPNQ in C_10_E_5_. One TPNQ binding event is observed in the mass spectrum. **b** Ion mobility mass spectrum indicates the 17^+^ charge state stabilizes the native-like state of the channel when bound to TPNQ. **c** Collision induced unfolding (CIU) plots for the 17^+^ charge state (left) apo and (right) TPNQ bound GIRK2. **d** Stabilization calculated from parameters defined by fitting GIRK2 (17^+^ and 18^+^) bound to TPNQ using the software program, PULSAR^32,55^. Reported are average and standard error of the mean (s.e.m.) from repeated measurements (*n* = 3) in kiloelectron volts (keV). Source data are provided as a Source Data file

Given the high-quality samples of GIRK2 revealed by MS above, we next explored if we could resolve binding of small molecules, such as toxins and drugs. The first molecule we considered was tertiapin, a 21 amino acid peptide isolated from honey bee venom and a potent inhibitor of GIRK2 and other IRKs^21^. We used the oxidation resistant form of tertiapin where Met13 has been mutated to Q (TPNQ) with a molecular weight of 2452 Da^54^. The mass spectrum of a mixture of TPNQ to GIRK2 at a 6:1 molar ratio in the detergent C_10_E_5_ revealed direct binding of one TPNQ molecule (Fig. 2a). Interestingly, the arrival time distributions differed significantly for apo and TPNQ bound GIRK2 (Fig. 2b). For example, the 17^+^ charge state had a more compact conformer for GIRK2•TPNQ, whereas the apo species was predominantly an extended, activated conformer of the protein (Fig. 2b). These results prompted us to perform a collision induced unfolding (CIU) experiment where gas-phase unfolding is monitored by IM, an approach that has been successfully employed for soluble^34^ and membrane protein complexes^32^. The IM profiles begin with a native-like arrival time distribution where energy regimes that are sufficient to remove detergent micelles but insufficient to perturb protein structure are used (Fig. 2c and Supplementary Fig. 7), as observed for other membrane protein complexes^32^. As collision energy is increased, the arrival time distribution transitions to a partially unfolded state (Fig. 2c and Supplementary Fig. 7) at 145 electron volts (eV) for the 17^+^ charge state of GIRK2. Since charge-reducing detergents are used, there is no dissociation of GIRK2 and GIRK2•TPNQ complexes even at the highest collision energies. Applying algorithms to quantify transitions in CIU profiles^32,55^, we calculated an average stabilization for the binding of TPNQ to GIRK2 of 640 and 637 eV for the 17^+^ and 18^+^ charge states, respectively (Fig. 2d). In summary, CIU experiments reveal that TPNQ bound GIRK2 significantly stabilizes the channel with an increased resistance to the gas-phase unfolding forces.

The second small molecule we studied was ivermectin, a hydrophobic drug molecule identified in functional assays to activate GIRK2 channels^24,56^. The drug has a molecular weight of 875 Da that is comparable to the mass of PIPs (Supplementary Fig. 8A). Unlike TPNQ, we observed no binding of ivermectin to the channel under our experimental conditions even at concentrations as high as 300 μM (Supplementary Fig. 8B), which is threefold higher than values used in previous studies. Since functional studies report a PI(4,5)P_2_-dependent but G_βγ_-independent activation of GIRK2 channels by ivermectin, we incubated GIRK2 with a mixture of ivermectin and a PI(4,5)P_2_ with 18:0–20:4 tails (PI(4,5)P_2_-sa) at 1:100:6 molar ratio, respectively. The mass spectrum for these mixtures compared to the mass spectrum to a sample free of ivermectin was indistinguishable, implying no direct binding of the drug (Supplementary Fig. 8c, d). In addition, no significant mass spectral peak broadening or shift was observed in the presence of the drug. These results demonstrate the ability of our MS approach to identify molecules that are direct binders.

### Selectively binding of lipids to GIRK2

**Fig. 3:**
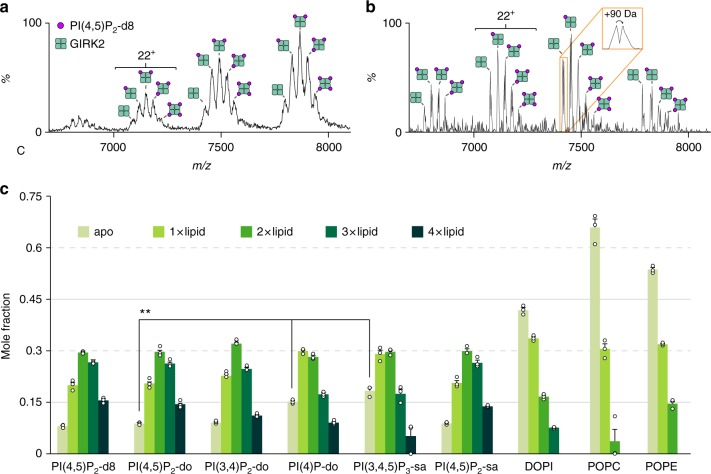
Selective binding of PIPs and phospholipids to GIRK2. Representative mass spectra of a mixture of 0.5 µM GIRK2 and 3 µM PI(4,5)P_2_-d8 acquired on the **a** Synapt G1 and **b** Exactive Plus EMR. Up to four lipid binding events are observed in the mass spectra, and higher resolution achieved by the EMR enabling small molecule adducts to be resolved. **c** Plot of mole fraction data obtained from deconvoluting mass spectra for various lipids mixed with GIRK2 at a molar ratio of 6:1, respectively. GIRK2 has much higher affinity for PIPs over other phospholipids. Reported is the average and s.e.m. (*n* = 3). Student’s *t* test (two-tailed) was used for statistical analysis (^**^P < 0.01) with the apo molar fraction being used for comparison. Lipid abbreviations are provided in Supplementary Table 3. Source data are provided as a Source Data file

Previous work suggests that native MS can accurately reflect the solution binding properties of proteins, small molecules, and lipids interacting with membrane proteins^33,39,40^. Therefore, we used native MS to probe the selectivity of PIPs. To this end, we incubated GIRK2 with a sixfold molar excess of phosphatidylinositol-4-phosphate (PI(4)P), phosphatidylinositol-3,4-bisphosphate (PI(3,4)P_2_), PI(4,5)P_2_ with dioleoyl acyl chains (18:1–18:1, do), dioctanoyl (8:0–8:0, d8) PI(4,5)P_2_, 1-stearoyl-2-arachidonoyl (18:0–20:4, sa) PI(4,5)P_2_-sa, and phosphatidylinositol-3,4,5-triphosphate (PI(3,4,5)P_3_-sa) (see Supplementary Table 3 for abbreviations). Native mass spectra for GIRK2 incubated with PIPs revealed binding of up to four molecules including peaks corresponding to the apo channel (Fig. 3). As the mass spectral peaks were broad, we introduced the sample into the REIS-Orbitrap and obtained higher resolution mass spectra with slightly less lipid-bound fractional abundances (Fig. 3b). We observed peak splitting in the presence of PI(4,5)P_2_-d8 corresponding to 90 ± 40 Da. After deconvoluting the mass spectra^57^ and extracting mole fraction data, PI(4,5)P_2_-do and PI(3,4)P_2_-do bound with similar abundance to PI(4,5)P_2_-d8 (Fig. 3c). In the case of PI(4)P-do, which contains one less phosphate group, the overall abundance of bound lipid was significantly reduced as evident by an increase in the fractional abundance of apo GIRK2. PI(3,4,5)P_3_-sa, a lipid with three phosphates groups on the inositol head group, also showed a reduction in binding as indicated by a higher fractional abundance of the apo channel compared to PI(4)P-do. The mass spectrum for PI(4,5)P_2_-sa, a lipid with similar acyl chains as PI(3,4,5)P_3_-sa, had comparable binding to the other acyl variants of PI(4,5)P_2_ studied. These data indicate GIRK2 selectively binds PI(3,4)P_2_ and PI(4,5)P_2_ with roughly equivalent affinity while displaying a lack of sensitivity toward acyl chain length.

We next examined the data to compare the gas-phase stabilization imparted by lipids and TPNQ. The ATD for PIP_1–4_ bound to GIRK2 acquired under optimized instrument settings did not preserve the native-like distribution for the 17^+^/18^+^ ions when compared to GIRK2 bound to TPNQ (Supplementary Fig. 9A, B). We then performed CIU on the apo and PI(4,5)P_2_-do bound to GIRK2 and found that a modest stabilization, between 180 and 230 eV, was achieved when four PI(4,5)P_2_-do were bound to GIRK2 (Supplementary Fig. 9). PIP binding clearly stabilizes the channel but to a lesser extent than TPNQ.

To compare the affinity of PIPs to other phospholipids we considered binding of PI with do-type acyl chains (DOPI), phosphatidylcholine (PC), and phosphatidylethanolamine (PE) with 1-palmitoyl-2-oleoyl (16:0–18:1, PO) acyl chains at the same molar ratio used for the PIP binding studies described above (Fig. 3c). In the case of DOPI, a precursor for PIPs, there was a significant reduction in binding and only up to three lipid binding events were observed compared to four PIPs bound. For POPE, a maximum of two lipids were bound, with 66% of the signal corresponding to apo GIRK2. POPC differs from POPE by three additional methyl groups on the choline head group and was the weakest binder of the lipids studied. Other studies have reported the sensitivity of Kir2 to PI(4,5)P2 can be modulated by anionic lipids^19^, we explored this possibility with GIRK2 and incubated the channel with four molar equivalents of PI(4,5)P2-do and POPG (Supplementary Fig. 10). The mass spectrum revealed no significant differences in binding of PI(4,5)P2-do compared to samples lacking POPG, which would be expected for competitive binding or positive allosteric modulation^42^. Taken together, GIRK2 binds PIPs with much higher affinity compared to other phospholipids.

### Lipid binding affinity for the R201A mutant of GIRK2

**Fig. 4:**
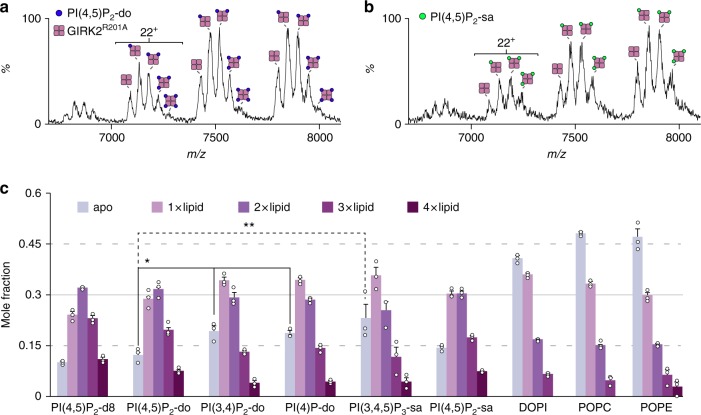
Altered selectivity of GIRK^R201A^ toward PIPs and phospholipids Representative mass spectra of GIRK2^R201A^ mixed with six equivalents of **a** PI(4,5)P_2_-do and **b** PI(4,5)P_2_-sa. **c** Mole fraction derived from mass spectrometry data for GIRK2 mixed with different lipids at sixfold molar excess. Reported is the average and s.e.m. (*n* = 3). Student’s *t* test (two-tailed) was used for statistical analysis (^*^P < 0.03) as described in Fig. 3. Source data are provided as a Source Data file

We then considered the GIRK2^R201A^ mutant, a channel reported to gate by PIPs independently of G_βγ_ and performed similar lipid binding experiments as done for the wild-type channel (Fig. 4). The most abundant binding was observed for PI(4,5)P_2_-d8 and binding was similar for PI(4,5)P_2_-do, and PI(4,5)P_2_-sa (Fig. 4c). In the case of PI(3,4)P_2_-do, a significant reduction in binding affinity was observed relative to wild-type GIRK2. The mass spectrum for PI(4,5)P_2_ and PI(3,4,5)P_3_ revealed comparable binding affinity to the wild-type protein. The mutant channel also displayed an overall increase in binding affinity toward DOPI, POPC and POPE compared to wild-type GIRK2. Taken together, GIRK2^R201A^ displays a greater selectivity towards PIP isoforms with head group phosphorylation at the 4 and 5 positions and reduced affinity toward the other PIPs.

In a similar fashion to the wild-type channel, we examined the GIRK and GIRK^R201A^ data to compare the gas-phase stabilization imparted by lipids and TPNQ. GIRK^R201A^ bound to TPNQ was significantly stabilized to a level that was comparable to the wild-type protein (Supplementary Figs. 11 and 12). Like the wild-type protein, PIPs had similar stabilization to the mutant channel under the optimized instrument settings used. Lastly, we compared charge states that represent partially unfolded and native-like states (the 21^+^ and 16^+^, respectively) (Supplementary Fig. 13A). Significant dissociation was observed for DOPI, POPC, and POPE at higher charge states (Supplementary Fig. 13B, C). In contrast, the abundance of PIPs bound were nearly identical between the two charge states suggesting that GIRK2 specifically bind PIPs. This phenomenon is supported by observation of increased binding affinity for PIPs in relation to other phospholipid species.

## Discussion

In order for a drug to be efficacious it must directly bind and modulate the function of the target protein^58,59^. Here, we demonstrate the utility of native MS to monitor direct binding of small molecules to GIRK2. The high-quality samples produced herein enabled native mass measurements of a eukaryotic ion channel with the ability to resolve adducts as small as 80 Da on the intact 156 kDa tetrameric complex. These adducts represent GIRK2 phosphorylation as shown by MS of intact, denatured GIRK2 and traditional bottom-up proteomics analysis. The covalent modifications mapped to surface exposed residues located in the CTD and, unexpectedly, to both serine residues within the linker region of the expression construct. Removal of the serine residues in the linker region will undoubtedly improve protein homogeneity. For GIRK2, the reported phosphorylation events that have roles in protein trafficking and modulating activity are located in the N-terminal region^2,60–62^. Although the GIRK2 phosphorylation sites we identified here warrant further study, it is important to highlight that a native mass spectrum can provide insight in protein modifications that may go undetected. Taken together, our MS results and methods provide the foundation for future studies to not only improve sample quality but also detect post-translational modifications.

The optimized instrument settings for GIRK2 resulted in a balance of compact, native-like arrival times for half of the charge states as determined by IM. Here, we have avoided interpreting activated ions and only deconvoluted regions of the mass spectrum corresponding to ions with native-like arrival times. Another potential issue is the fact that electrostatic and hydrophobic interactions can be altered in the gas-phase. However, in our previous reports^33,39^, and from the Klassen group^40^, binding thermodynamics for soluble protein-ligand, membrane protein–protein, and membrane protein–lipid interactions determined by native MS are in direct agreement with SPR and ITC. The fact that similar equilibrium constants and thermodynamic parameters to solution measurements can be determined by native MS suggest the altered electrostatic and hydrophobic interactions in the gas-phase are negligible. Although we have not corroborated our lipid binding data with another assay, IM measurements allow us with confidence to extract mole fraction lipid binding data for ions with compact, native-like arrival times and minimize potential gas-phase artifacts.

Native IM MS not only can monitor direct binding events but probe membrane protein stability in complex with small molecules. The IM–MS data for GIRK bound to TPNQ provide compelling evidence that the toxin binds and significantly stabilizes the channel. Increased protein stabilization upon ligand binding, including membrane protein–lipid interactions, has been well characterized by IM^32,34^. Notably, the gas-phase stabilization observed here for GIRK2•TPNQ is over sixfold more stabilizing compared to values reported for membrane protein–lipid interactions for a single binding event^32^. In the case of the recently reported GIRK2 activator ivermectin, no direct binding was observed. The absence of ivermectin binding to GIRK2 using our native IM–MS approach suggests the drug molecule may bind weakly and/or require a lipid bilayer to support binding. Another possibility for no apparent binding is the energy regime required to release the protein from the detergent micelle is too high and may dissociate the bound drug.

Native IM–MS data reveal that both GIRK2 and GIRK2^R201A^ display distinct specificity for the phosphorylation pattern of the inositol head group. More specifically, GIRK2 and GIRK2^R201A^ both have high affinity for PI(4,5)P_2_ whereas the wild-type channel also has comparable affinity for PI(3,4)P_2_. Although the majority of assays use predominantly PI(4,5)P_2_ to activate GIRK2, our results are largely in agreement with functional studies for other GIRKs, which observed head group selectivity compared to other IRKs^16,19,27^. The slight differences between our direct binding measurements and channel activation represents an interesting question as to the correlation between PIP binding events and gating. Moreover, the acyl chain variations among the PIPs explored here show insignificant differences in preference that is in contrast with functional studies that reported differences in acyl chains lengths for GIRK1/4^16^. Although GIRK2 is in the same subfamily as GIRK1/4, the data suggests these two channels have a different selectivity towards the lipid tails of PIPs. In summary, GIRK2 lipid selectivity is largely determined by head group phosphorylation pattern of the PIPs, and not necessarily through acyl chain length.

MS results reveal that GIRK2 and GIRK2^R201A^ bind PIPs cooperatively and with much higher affinity than highly abundant phospholipids in the plasma membrane^63^. GIRK2 binds DOPI, POPC, and POPE with reduced binding affinity compared PIPs. At equimolar concentrations, up to three lipid binding events to GIRK2 were observed for DOPI, POPC, and POPE, whereas four PIPs can be seen bound. The much higher binding affinity of GIRK2 toward PIPs provides evidence for how these channels can bind PIPs that are found in low abundance compared to bulk lipids in the plasma membrane.

Allosteric modulation of protein function by binding of molecules at remote sites is an important biological phenomenon^64–66^. Here, GIRK2^R201A^ allowed us to explore lipid binding to a channel that induces a rotation in the CTD, similarly to G_βγ_-activated state of GIRK2^25^. The mutant was primarily used because the biochemical isolation of the GIRK2•G_βγ_ complex in detergent solutions has so far been unsuccessful^26^. Early studies indicate GIRK interactions with PI(4,5)P_2_ are weak and that there is an increase in relative affinity for the G_βγ_-activated channel^9,67^. However, our data suggest that rotation of the GIRK2 CTD when bound to G_βγ_, as mimicked here by GIRK2^R201A^, does not enhance or decrease the binding affinity toward PI(4,5)P_2_. Rather, rotation of the CTD allosterically modulates remote PIP binding sites, tuning the channel by reducing the binding affinity for PIP isoforms known to weakly or not activate GIRKs. In essence, the reduction in binding affinity for specific PIP isoforms promotes interaction with PI(4,5)P_2_, a potent GIRK2 activator. Our findings are in-line with recent MS studies suggesting membrane protein–lipid interactions can allosterically modulate interactions with regulatory and signaling proteins^39,68^. Although the R201A CTD rotation is greater than the GIRK2•G_βγ_ complex, we propose that rotation of the CTD allosterically tunes the selectivity of the channel toward PIPs.

## Methods

### Protein expression and purifications

Mouse GIRK2 was expressed and purified from KM71 *Pichia pastoris* (Invitrogen) following established methods with considerable modifications^25^. In brief, the pPICZ vector to expresses chicken Kir2.2 was a generous gift from Dr. Roderick MacKinnon and subsequently modified to have a tobacco etch virus (TEV) protease cleavable C-terminal green fluorescent protein (eGFP) followed by a StrepII tag and 6×His-tag. The GIRK2 (KCNJ6) gene from Mus musculus (residues 52–380) was codon optimized for *Pichia pastoris* using the Codon Optimization Tool from Integrated DNA Technologies (IDT) and synthesized as a gBlocks gene fragment (IDT). An In-Fusion cloning kit (Clonetech) was used to clone the codon optimized GIRK2 gene fragment containing appropriate vector overhang sequences into the modified pPICZ vector linearized with XhoI and EcoRI (New England BioLabs). The R201A mutant was generated using a sited-directed mutagenesis kit (Agilent). Complete list of primers and plasmids used in this study are provided in Supplementary Table 4. *P. pastoris* were transformed with PmeI linearized plasmids and the highest expressing clones selected based on eGFP expression levels. These clones were expanded, harvested and lysed using a microfluidizer (Microfluidics Corporation). The clarified lysate was supplemented with DDM to extract membrane proteins. Ni-NTA agarose resin and StrepTactin sepharose resin were used in tandem to purify the GIRK2 fusion protein followed by processing with TEV protease to remove the C-terminal tag. GIRK2 was treated with a DHPC wash to remove the remaining contaminants and then detergent-exchanged into C_10_E_5_ by size exclusion chromatography. The peak fractions were pooled and snap-frozen in liquid nitrogen and stored at −80 °C until needed. Additional details can be found in Supplementary Methods.

### Sample preparation for native MS

Lipids were prepared^33^ by dissolving in chloroform, removal of chloroform with a stream of nitrogen gas to generate a film and dried by desiccation. Lipid films were resolubilized in MS buffer (100 mM ammonium formate, 0.065% C_10_E_5_, and pH 7.2 at room temperature). TertiapinQ (Alomone Labs) was directly solubilized in MS-compatible buffer. Ivermectin was first solubilized in DMSO, then diluted into MS buffer supplemented with ethanol to maintain solubility. GIRK2 was buffer exchanged into 100 mM ammonium formate (pH 7.3) supplemented with either 0.065% C_10_E_5_ (w/v) or 0.022% (w/v) DDM by centrifugal desalting columns (BioRad). Gold-coated capillary tips were prepared by first pulling borosilicate capillaries (Cat no., Sutter Instruments) with a flaming/brown micropipette puller (P-1000, Sutter Instruments) followed by coating with gold using a sputter coater (EM ACE200, Leica Microsystems)^44^. All mixtures of lipids/ligands with protein were allowed to incubate at room temperature for 2 min and loaded into a gold-coated capillary tip. Additional details can be found in Supplementary Methods.

### MS analysis

In short, native MS was performed on a Synapt G1 HDMS instrument (Waters Corporation) equipped with a 32k RF generator or on a Thermo Scientific Exactive Plus extended mass range (EMR) with a rear-entry ion source (REIS)^49^. IM data were processed using PULSAR^55^. Mole fraction was determined from deconvolution of mass spectra with UniDec^57^. Denatured MS was performed on the front end of the REIS-orbitrap. Tryptic digest analysis and protein sequencing was performed on Thermo Orbitrap Fusion. Further details can be found in Supplementary Methods.

### Reporting Summary

Further information on experimental design is available in the Nature Research Reporting Summary linked to this article.

## Supplementary information

Supplementary Information

Reporting Summary

Source Data File

## Data Availability

Data supporting the findings of this manuscript are available from the corresponding author upon reasonable request. A reporting summary for this Article is available as a Supplementary Information file. Bottom-up proteomics data has been deposited at proteomeXchange with accession code PXD012894. The source data underlying Figs. 1a–d, 2a–d, 3a–c, 4a, c and Supplementary Figs. 1a–c, 2a, b, 3a, b, 4, 8b–d, 9e, 10a, b, 11, and 13b, c are provided as a Source Data file.
