# Supplementary material for: Selective binding of a toxin and phosphatidylinositides to a mammalian potassium channel
Source: Nat Commun. 2019 Mar 22;10:1352. doi: 10.1038/s41467-019-09333-4 (PMC6430785; doi:10.1038/s41467-019-09333-4)
Supplement: Supplementary file 1 — Supplementary Information [file 41467_2019_9333_MOESM1_ESM.pdf]

Selective Binding of a Toxin and Phosphatidylinositides to a Mammalian Potassium Channel

Yang Liu[1], Catherine E. LoCaste[2], Wen Liu[1], Michael L. Poltash[2], David H. Russell[2], Arthur Laganowsky[2,*]

[1]Institute of Biosciences and Technology, Texas A&M Health Science Center, Houston, TX 77030

[2]Department of Chemistry, Texas A&M University, College Station, TX 77842

Supplementary Methods

Construct preparation and expression of recombinant mouse GIRK2 in *Pichia pastoris*

For construction of the GIRK2 expression plasmid, the KCNJ6 gene from *Mus musculus* (amino acid 52-380 used for structure determination[1]) was codon optimized for *Pichia pastoris* using the Codon Optimization Tool from Integrated DNA Technologies (IDT) and synthesized as a gBlocks gene fragment (IDT). The pPICZ vector to express chicken Kir2.2 was a generous gift from Dr. Roderick MacKinnon and subsequently modified to have a C-terminal StrepII tag and 6xHis-tag preceded by eGFP to facilitate protein purification. This vector was also modified to contain a TEV (Tobacco Etch Virus) protease site between the protein of interest and the eGFP-StrepII-6xHis tag. In-Fusion cloning kit (Clonetech) was used to clone the codon optimized GIRK2 gene fragment into the modified pPICZ vector linearized with XhoI and EcoRI (New England BioLabs). This produced a construct which can express GIRK2-eGFP-StrepII-6xHis fusion protein under the control of the inducible alcohol oxidase promoter with eGFP used as a reporter of protein expression. The R201A mutant was generated using the QuikChange Lightning Site-Directed Mutagenesis Kit (Agilent) following manufacturer's protocol. The resulting plasmids were verified by restriction digest analysis and DNA sequencing. Complete list of primers and plasmids used in this study are provided in Supplementary Table 4.

The GIRK2-eGFP-StrepII-6xHis containing plasmid was linearized with PmeI (New England BioLabs) and transformed into *P. pastoris* KM71 by electroporation. Transformants were selected on YPD plates containing 0.6 M sorbitol, 500 to 1,000 µg/mL zeocin and 2% agar. The *P. pastoris* integrant colony which showed highest eGFP level (*i.e.* greenest color) during small-scale expression screening (15 mL culture after 30 hours post induction with methanol) was used for large-scale expression. To produce starter culture, a single colony of *P. pastoris* GS115/pPICZB-GIRK2-eGFP-StrepII-6xHis was grown (27 °C, 220 rpm) for 20 hours in BMGY media (1.34% yeast nitrogen base, 1% yeast extract, 2% peptone, 0.1 M potassium phosphate, pH 6.1, 1% glycerol, 0.4 µg/ml biotin, and 40 µg/mL histidine) containing 150 µg/mL zeocin. Cells were pelleted (1,500 g, 10 min) and then resuspended in BMMY media (BMGY medium without glycerol but with 1% methanol) for induction with methanol. Cultures were grown (27 °C, 220 rpm) for 48 hours with addition of 0.5% methanol twice daily. Cells were pelleted (1,500 g, 10 min), resuspended in wash buffer (50 mM 2-amino-2hydroxymethyl-propane-1,3-diol [Tris], 150 mM NaCl, pH 7.4), and then pelleted again. Cell pellets were stored at -80 ºC and typically used within 2 weeks.

Protein Expression and Purification

Frozen *P. pastoris* pellets were thawed and resuspended in lysis buffer (300 mM KCl, 50 mM Tris, pH 7.4 at room temperature) at a ratio of 1:4 cell weight to buffer volume ratio. The cell mixture was supplemented by a protease inhibitor cocktail tablet (Roche Pharmaceuticals) and was stirred until homogenized. The mixture was passed through an M-110PS microfluidizer (Microfluidics) at 30,000 psi for a total of four passes. Insoluble material was pelleted by centrifugation at 25,000 g for 30 min at 4 ºC. Supernatant was pooled and a final concentration of 2% DDM (n-Dodecyl-β-D-Maltopyranoside, Anatrace) was applied to the mixture to extract membrane proteins while stirring at 4 ºC overnight. Imidazole was then added to final concentration of 10 mM, and the mixture was clarified by centrifugation (100,000 g, 10 ºC for 30 min). The supernatant was then loaded onto an XK16/20 column (GE Healthcare) packed with 15mL of Ni-NTA Agarose beads (Qiagen) pre-equilibrated by buffer A (150 mM KCl, 30 mM Tris, 10 mM imidazole, 10% glycerol and 0.025% DDM, pH

7.6 at room temperature). After the solution was passed through the column, 15 mL of wash buffer consisting of buffer A supplemented with an additional 0.5% DDM was applied. The column was then exchanged into several column volumes of buffer A or until a steady baseline for 280 nm absorbance was established. The protein was eluted with 40 mL of buffer B (150 mM KCl, 30 mM Tris, 300 mM imidazole, 10% glycerol and 0.022% DDM, pH 7.6 at room temperature). Peak fractions were pooled, then loaded onto a HiPrep 26/10 desalting column (GE Healthcare) pre-equilibrated with Buffer C (150 mM KCl, 30 mM Tris, 10 mM β-mercaptoethanol (BME), 10% Glycerol and 0.022% DDM, pH 7.5 at room temperature). Peak fractions were pooled and then loaded onto two 5-mL StrepTrap HP Columns (GE Healthcare) connected in tandem pre-equilibrated in buffer C. Buffer D (150 mM KCl, 30 mM Tris, 10 mM BME, 4 mM d-desthiobiotin, 10% glycerol and 0.022% DDM, pH 7.5 at room temperature) was applied to elute recombinant protein from the StrepTrap HP Columns. Peak fractions were pooled and once again loaded onto a HiPrep 26/10 desalting column pre-equilibrated with Buffer C to remove d-desthiobiotin. Peak fractions were pooled before adding His-tagged TEV protease produced in-house [2]. The mixture was incubated overnight at 9 ºC and then filtered through a 0.45 µm syringe filter (Pall Corporations). The protein solution was loaded onto a 5-mL HisTrap HP column (GE Healthcare) equilibrated in buffer E (150 mM KCl, 30 mM Tris, 25 mM imidazole, 10% glycerol and 0.022% DDM, pH 7.6 at room temperature). Flow-through containing the untagged GIRK2 was collected and concentrated using a 100,000 MWCO concentrator (Millipore) to roughly 2 mg/ml as determined by UV absorbance (with coefficient of 1 Abs = 1 mg/mL). The protein was flash-frozen in liquid nitrogen, stored at -80 ºC, and typically used within two weeks. These steps, including the desalting step following the StrepTrap, are crucial for the stability of the protein prior to the final polishing step for mass spectrometry analysis.

GIRK2 Polishing for Native Mass Spectrometry Studies

About ~1 mg of purified GIRK2 protein solution was thawed and added to buffer F (150 mM KCl, 50 mM NaCl, 30 mM Tris, 10 mM BME, 1 mM DTT [1,4-dithiothreitol], 10% glycerol, 15 mM DHPC [1,2-diheptanoyl-sn-glycero-3-phosphocholine] and 0.022% DDM, pH 7.5 at room temperature). The ratio of protein solution to buffer F added is 1:8 by volume and the solutions were kept at 15 ºC before mixing. The mixture was immediately concentrated in a 100,000 MWCO concentrator at 3,000 g and 15 ºC for 3 minutes, and the mixture was manually re-suspended with a pipette. This process was repeated until 1 mL of solution remain to avoid precipitation of the protein. 10 mL of buffer C was added to the concentrator and the sample slowly concentrated to a final volume of 500 µL with repeated resuspension of the solution in the concentrator every 3 minutes to minimize aggregation. The mixture was then filtered through a 0.22 µm spin-filter (Millipore) before being injected into a Superdex 200GL 10/300 (GE Healthcare) column equilibrated in buffer G (150 mM KCl, 50 mM NaCl, 30 mM Tris, 10% glycerol, 0.07% $C_{10}E_5$ (decylpentaglycol, Anatrace), and pH 7.4 at room temperature). Peak fractions containing delipidated GIRK2 were pooled and concentrated in a 100,000 MWCO concentrator until 50 µL total volume was reached. Concentrated proteins were either flash-frozen in liquid nitrogen and stored at -80 ºC, or used directly by exchanging into MS buffer (100 mM ammonium formate, 0.065% $C_{10}E_5$, and pH 7.2 at room temperature) using a centrifugal buffer exchange device (MicroBio-Spin6, Bio-Rad) following manufacture's protocol. Ammonium formate is used instead of the typical ammonium acetate due to GIRK2 having slightly better shelf-life in this buffer. For convenience, the sample in MS buffer was aliquoted into 2 µL fractions, flash-frozen in liquid nitrogen and stored at -80 ºC until used for experiments. Notably, we found no observable difference in mass-spectral quality after a single freeze-thaw of GIRK2 proteins up until this point of our purification regime.

Preparation of Phospholipids and Other Ligands for MS Binding Studies

Phospholipid stock solutions were prepared as previously described.[3] Briefly, chloroform was removed from the phospholipid ampoules, which were pre-aliquoted by Avanti Polar Lipid, by gentle nitrogen gas flow and then by desiccation overnight. For lipids purchased in powder form, chloroform was added, and the solution was transferred to a new glass vial and dried as described above. The dried lipid films were solubilized in the MS buffer. Lipid concentrations were calculated directly from the weight of each aliquots in individual vials.

The molar ratio of wild-type or R201A GIRK2 to phospholipids was held constant at a ratio of 1:6. Protein aliquots were kept on dry ice until mixing with lipid solution at a 1:1 volume ratio. Specifically, GIRK2 and lipid mixtures were held at a final concentration of about 500 nM and 3 μM, respectively. The R201A GIRK2 and lipid mixtures were at a final concentration of about 825 nM and 5 μM, respectively. These protein-lipid mixtures were allowed to incubate for 2 minutes at room temperature before loading into a gold-coated glass capillary tip produced in-house as previously described.[4] We have found that longer incubation time does not change the mole fractions of apo and lipid-bound proteins. These findings are in agreement with our previous studies for other membrane proteins.[3]

Ivermectin (Alfa Aesar) was solubilized in DMSO and/or 100% ethanol, and dilutions made in MS buffer. No noticeable precipitation of ivermectin was observed after dilution in MS buffer until a final concentration of 2mM in 20% DMSO or 5% ethanol, 3% DMSO is reached. This sample was mixed with GIRK2 in a 1:5 ratio by volume of ivermectin to GIRK2. Tertiapin Q (TPNQ, Alomone Labs) was diluted directly in MS buffer to 100 μM or lower, and mixed with GIRK2 in a 1:1 ratio by volume.

Native Mass Spectrometry and Data Analysis

A Synapt G1 HDMS instrument (Waters Corporation) with a 32k RF generator was used for most of the data collected. Instrument parameters were tuned to maximize ion intensity and simultaneously preserve the native-like state of GIRK2. The capillary voltage was set to1.75 kV, sampling cone voltage at 200 V, extractor cone voltage at 10 V and argon flow rate at 7 mL/min (5.2 x 10^{-2} mbar). The T-wave settings for trap (300 ms and 1/1.0 V), IMS (300 ms and 1/18 V) and transfer (100 ms and 1/10 V), source temperature (90 ºC) and trap bias (35 V) were also optimized. Trap and Transfer Collision Voltage were set to 100 V and 60 V, respectively, unless otherwise noted. Ion mobility mass spectrometry data were processed using the software program Pulsar [5] and de-convoluted using UniDec[6] followed by converting intensities of GIRK2 and PIP-GIRK2 species to mole fractions as described previously.[3]

High-resolution MS spectra were collected using an Exactive Plus with extended mass range (EMR) from Thermo Scientific. Samples were analyzed using both the original instrument and a modified reverse entry ion source (REIS) coupled to the HCD cell of the Orbitrap.[7] Gold-coated capillaries described above were loaded with sample. Operating conditions of the REIS were described in detail previously; briefly, ions are generated by nano-ESI, focused with a RF ion funnel, and transferred to the HCD cell by an octupole ion guide. All mass spectra were collected with 10 µscans at 17 500 mass resolution with an ion injection time of 200 ms. Collision energies of 20 eV in-source CID and 70 HCD collision energy for normal operating conditions and 110 HCD collision energy for REIS. These energies were chosen to effectively desolvate and strip detergents while minimizing perturbations to protein-lipid and subunit interactions. Native MS data from the Exactive Plus EMR is analyzed and mass species assigned using UniDec.[6]

Top-down and Bottom-up Mass Spectrometry

A sample of denatured GIRK2 was prepared using method described by Campuzano and co-workers[8] with minor modifications. A TSKgel Phenyl-5PW RP column (7.5 cm x 4.6 mm, Tosoh Bioscience) was connected to an AKTA Avant (GE Healthcare) an equilibrated in 30% n-propanol, 0.1% Formic Acid (FA), and 0.1% Trifluoracetic acid (TFA). An aliquot of GIRK2 with purification tags removed by TEV protease was incubated with 5 mM BME prior to injection onto the column. The denatured GIRK2 protein was eluted with a gradient over three mL to 100% n-propanol, 0.1% FA, and 0.1% TFA at a flow rate of 1 mL/min. The peak fraction containing denatured GIRK2 was directly infused into the front-end source of Exactive Plus EMR instruments with the following settings: Scan resolution was set to 17 500, 100 eV in-source CID and 10 HCD collision energy was used, as well as 125 degrees ºC for capillary temperature. For bottom-up analysis, TEV-processed GIRK2 was digested with trypsin overnight, then applied through a liquid chromatography electrospray ionization tandem mass spectrometry (LC-ESI-MS/MS) system, consisting of a Dionex Ultimate

3000 LC system (with an Acclaim PepMap 100 C18 column from Thermo) coupled to a Thermo Orbitrap Fusion mass spectrometer. LC system solvents were water + 0.1% FA (A) and acetonitrile with 0.1% FA (B). Tryptic peptides were eluted over 60 minute gradient from 2% B from 0 to 5 min, 2% to 45% from 5 to 37 min, 45% to 90% from 38 to 46 min, and down to 2% from 46 to 60 min at a flow rate of 0.4 uL/min. The mass spectrometer ion source was set to have a spray voltage of 2.3 kV, ion transfer tube temperature of 275 ºC, the scan range was m/z 400–1600 with a resolution of 120,000. MS/MS acquisition was performed with 3 s cycle time. The intensity threshold was set to 5000, Ions with charge states 1+ to 6+ were sequentially fragmented by high energy collisional dissociation (HCD) with a normalized collision energy (NCE) of 28%. The dynamic exclusion duration was set as 60 s. Raw files were analyzed using the Thermo Proteome Discoverer (v2.1.0.81) software platform. The mass spectrometry data was analyzed using SEQUEST with the following parameters: the protein sequence database contained only the recombinant GIRK2 sequence, trypsin selected as the enzyme, dynamic (or variable) modifications included protein N-terminal acetylation, oxidation, serine, threonine and tyrosine phosphorylation. Carbamidomethylation of cysteine was set as a fixed modification since trypsin digested samples was treated with iodoacetamide. Mass tolerances for precursor ions was set to 10 ppm, and fragment ions set to Δ0.6 Da. Limits for peptide length searched range from 6 to 144, maximum delta Cn is set to 0.05. Maximum number of allowed missed cleavages is 2.

Supplementary Tables

Supplementary Table 1. Calculated and measured masses for GIRK2^{R201A} and GIRK2 bound to different ligands acquired on a Synapt G1 instrument. Measured mass was determined by deconvolution of native mass spectra using the program, UniDec.[6] For ligands bound to GIRK2, the addition of mass to the apo GIRK2 mass is reported. The reported standard deviation was calculated directly from the width of the zero-charge mass spectrum from UniDec after deconvolution.

Species	Calculated Mass (Da)	Measured Mass (Da)
GIRK2	156,292	156,296 ± 230
GIRK2 bound to:		
Tertiapin Q	Δ2,452	Δ2,450 ± 231
Ivermectin	Δ875	–
PI(4,5)P_2-d8	Δ798	Δ740 ± 320
PI(4,5)P_2-do	Δ1,074	Δ1,080 ± 351
PI(3,4)P_2-do	Δ1,074	Δ1,060 ± 353
PI(4)P_2-do	Δ977	Δ922 ± 372
DOPI	Δ880	Δ920 ± 361
PI(3,4,5)P_3-sa	Δ1,195	Δ1,060 ± 379
PI(4,5)P_2-sa	Δ1,098	Δ1,020 ± 375
POPC	Δ760	Δ802 ± 281
POPE	Δ718	Δ701 ± 357
GIRK2^{R201A}	155,952	155,989 ± 193

Supplementary Table 2. Bottom-up MS analysis of GIRK2 digested with trypsin identifies phosphorylation sites. The annotated sequences for phosphorylated fragments are shown. The covalently modified residues are shown in lower case. Reported are the measured masses, Δ ppm calculated from theoretical masses, retention time (RT), cross correlation values (XCorr), Delta score and Delta Cn from SEQUEST. Only GIRK2 sequence was used for the search. *Denotes the modified residue is within the linker region of the GIRK2 expression construct.

Annotated Sequence	Modifications	Mass [Da]	Δ ppm	RT [min]	XCorr	# Missed Cleavages	Delta Score	Delta Cn
FTPVLTMEDGFYEVDyNSFHETYETSTPSLSAK	Y353 (Phospho)	3885.71	7.51	26.75	4.12	0	0.01	0
FTPVLTMEDGFYEVDYNSFHETYEtSTPSLSAK	T362 (Phospho)	3885.71	7.51	26.75	3.94	0		0.04
FTPVLTMEDGFYEVDYNsFHETYETSTPSLSAK	S355 (Phospho)	3885.71	7.51	26.75	4.03	0		0.02
FTPVLTMEDGFYEVDYNSFHEtYETSTPSLSAK	T359 (Phospho)	3885.71	7.51	26.75	4.07	0		0.01
AQLPKEELEIVVILEGIVEATGMtcQAR	T320 (Phospho) C321 (Carbamidomethyl)	3177.59	-1.24	36.90	4.23	1	0.17	0
AESNsENLYFQ	S383 (Phospho)*	1381.52	-4.60	23.22	2.72	0	0.02	0
AESNsENLYFQ	S383 (Phospho)*	1381.52	-4.30	23.19	2.77	0	0.01	0
AESNsENLYFQ	S383 (Phospho)*	1381.53	-3.33	24.36	2.54	0		0.04
AEsNSENLYFQ	S381 (Phospho)*	1381.52	-4.60	23.22	2.66	0		0.02
AEsNSENLYFQ	S381 (Phospho)*	1381.53	-3.33	24.36	2.64	0	0.04	0
AEsNSENLYFQ	S381 (Phospho)*	1381.53	-1.38	25.95	2.03	0	0.07	0
AEsNSENLYFQ	S381 (Phospho)*	1381.52	-5.09	27.51	2.66	0	0.11	0
AEsNSENLYFQ	S381 (Phospho)*	1381.52	-4.30	23.19	2.75	0		0.01
ELAELANRAESNsENLYFQ	S383 (Phospho)*	2277.99	-5.40	25.98	3.10	1	0.02	0
ELAELANRAESNsENLYFQ	S383 (Phospho)*	2277.99	-4.36	25.11	2.73	1	0.17	0
ELAELANRAESNsENLYFQ	S383 (Phospho)*	2277.99	-3.88	33.66	2.71	1	0.26	0
ELAELANRAESNsENLYFQ	S383 (Phospho)*	2277.99	-5.89	23.86	4.71	1	0.04	0
ELAELANRAESNsENLYFQ	S383 (Phospho)*	2278.00	-2.83	28.57	3.03	1	0.10	0
ELAELANRAESNsENLYFQ	S383 (Phospho)*	2277.99	-5.08	24.94	3.19	1	0.12	0
ELAELANRAESNsENLYFQ	S383 (Phospho)*	2277.99	-4.76	30.30	3.31	1	0.06	0
ELAELANRAESNsENLYFQ	S383 (Phospho)*	2277.99	-4.55	31.79	3.06	1	0.19	0
ELAELANRAESNsENLYFQ	S383 (Phospho)*	2277.98	-7.98	23.86	5.92	1	0.08	0
ELAELANRAEsNSENLYFQ	S381 (Phospho)*	2277.99	-3.69	35.54	2.13	1		0.03
ELAELANRAEsNSENLYFQ	S381 (Phospho)*	2277.99	-5.89	23.86	4.49	1	0.05	0
ELAELANRAEsNSENLYFQ	S381 (Phospho)*	2277.99	-3.88	33.66	2.71	1	0.26	0

Supplementary Table 3. Full name and abbreviations for lipids used in this study.

Lipid	Abbreviation	Acyl Chains
1,2-dioctanoyl-phosphatidylinositol-4',5'-bisphosphate	PI(4,5)P_2-d8	08:0-08:0
1,2-dioleoyl-phosphatidylinositol-4',5'-bisphosphate	PI(4,5)P_2-do	18:1-18:1
1,2-dioleoyl-phosphatidylinositol-3',4'-bisphosphate	PI(3,4)P_2-do	18:1-18:1
1,2-dioleoyl-phosphatidylinositol-4'-phosphate	PI(4)P_2-do	18:1-18:1
1,2-dioleoyl-phosphatidylinositol	DOPI	18:1-18:1
1-stearoyl-2-arachidonoyl- phosphatidylinositol-3',4',5'-bisphosphate	PI(3,4,5)P_3-sa	18:0-20:4
1-stearoyl-2-arachidonoyl- phosphatidylinositol-4',5'-bisphosphate	PI(4,5)P_2-sa	18:0-20:4
1-palmitoyl-2-oleoyl-phosphatidylcholine	POPC	16:0-18:1
1-palmitoyl-2-oleoyl-phosphatidylethanolamine	POPE	16:0-18:1

Supplementary Table 4. List of primers and plasmids used in this study. *Deposition number for plasmid deposited at Addgene.

TevST2_F	GTCGAATTCTGAAAACCTGTATTTTCA GGGTTCCGGTAGCACTGCTGCCGCCGCTGTGAGCAAG
TevST2_R	TGTTCTAGACTATTACTTTTCGAACTG CGGGTGGCTCCAGCTTGCACCACCTCCGGACTTGTACAGC
6xHis_F	CACCATCATCACTAATAGTCTAGAACAAAAACTCATC
6xHis_R	GTGGTGGCTACCCTTTTCGAACTGCGGGTGGCTCCAG
mGIRK2_R201A_F	gaaaatttctcagcccaaaaaggcggctgaaactcttgttttctc
mGIRK2_R201A_R	gagaaaacaagagtttcagccgcctttttgggctgagaaattttc
pPICZ-mouseGIRK2-eGFP-StII-6xHis	124277*
pPICZ-mouseGIRK2R201A-eGFP-StII-6xHis	124278*

Supplementary Figures

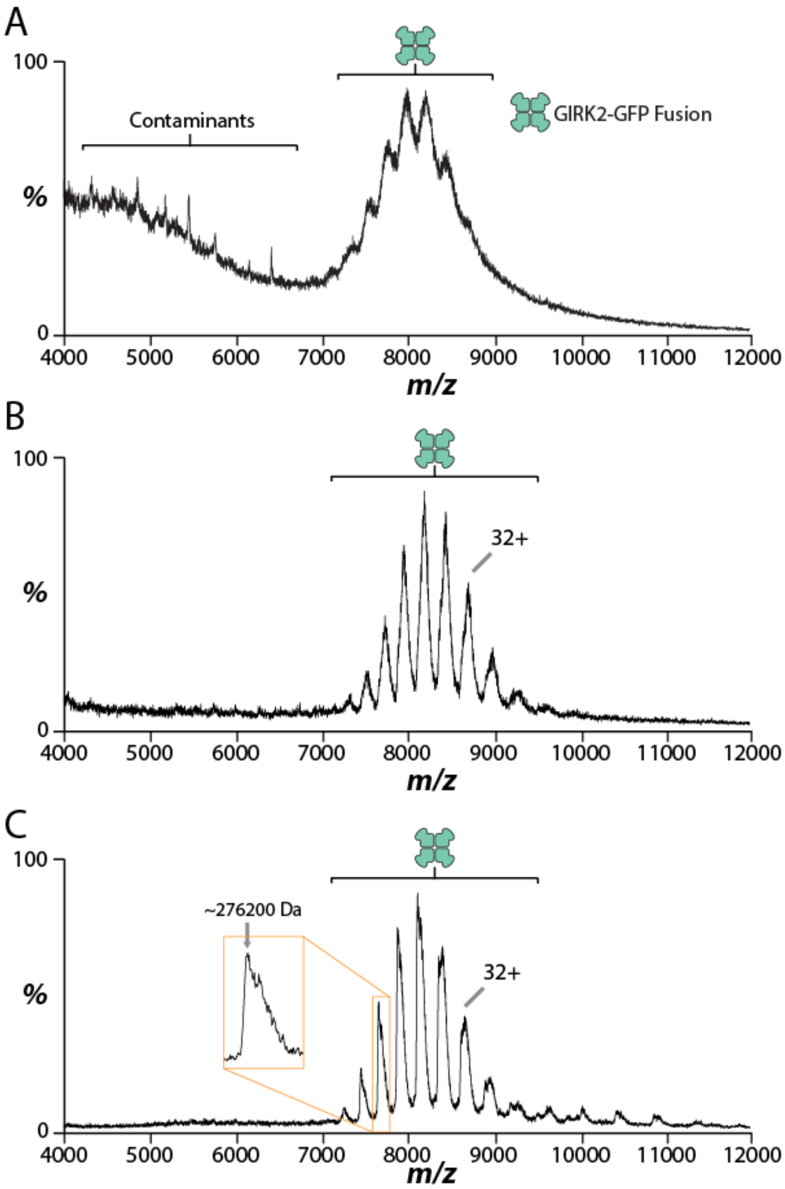

Supplementary Figure 1. Representative mass spectra of the GIRK2-GFP fusion protein showing the result of detergent screening performed to optimize sample quality for the GIRK2-GFP fusion protein. (A), Initial preparation of GIRK2 extracted in 3% DDM and analyzed in MS buffer containing 0.05% DDM, in a similar fashion to the established protocol for structural studies [9] but without performing size-exclusion chromatography (SEC). The mass of the tetrameric complex cannot be ascertained due to heterogeneous adduct species totaling at least several thousand Da, causing the significant broadness of peaks. Some contaminant proteins that were co-purified were visible on the left side of the spectrum. (B) GIRK2-GFP purified in 4% NG (n-Nonyl-β-d-glucoside, Anatrace), followed by SEC and MS analysis in buffers containing 0.05% DDM. While still difficult to accurately determine, the mass of the complex is much closer to the calculated average as bound adducts were reduced significantly. (C) GIRK2-GFP purified in 4% DDM, followed by buffer A wash supplemented with 2% NG, then analyzed in MS buffer containing 0.05% DDM. The combinatorial effect of both DDM and NG washes allowed sufficient removal of adducts such that the apo-GIRK2-GFP fusion protein is revealed at roughly 276.2 kDa.

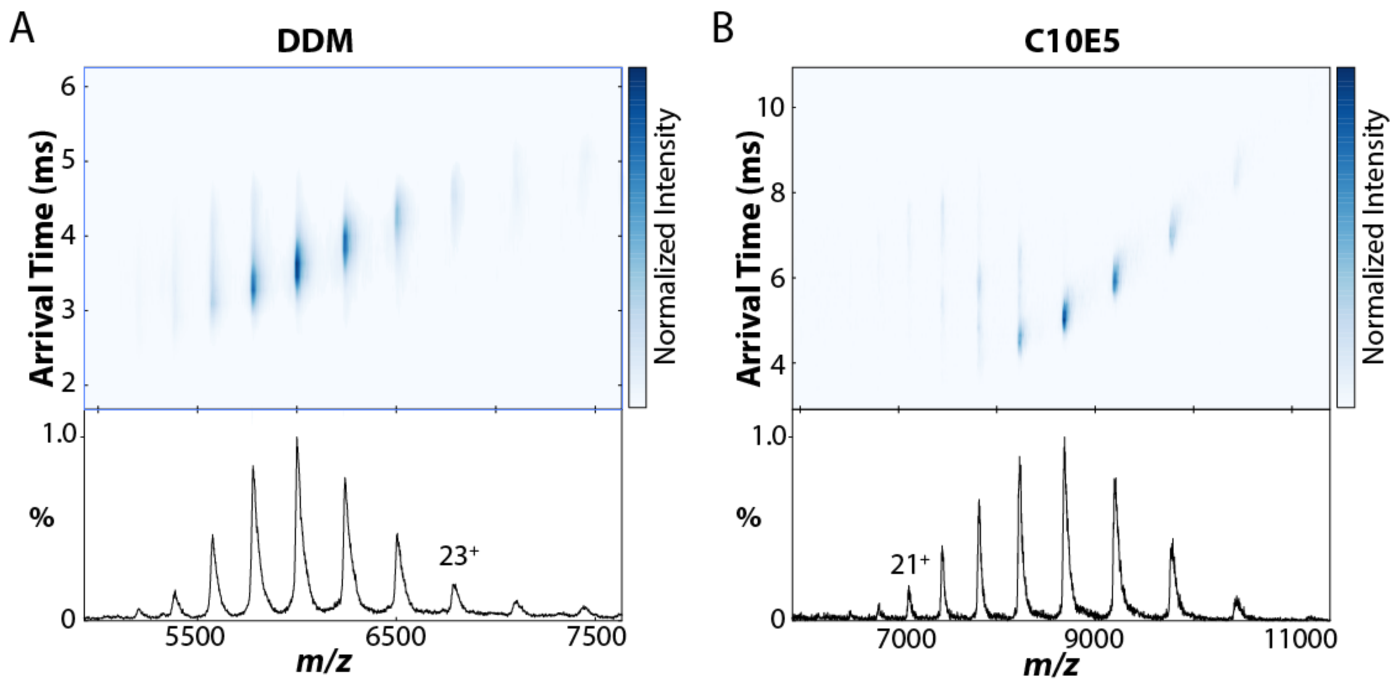

Supplementary Figure 2. Representative ion mobility mass spectra of GIRK2 tetramer (~156.2 kDa). GIRK2 in MS buffer containing 2x critical micelle concentration (CMC) of (A) DDM or (B) $C_{10}E_5$. The average charge state of GIRK2 in DDM buffer is about 26^+ whereas in $C_{10}E_5$ the average is reduced to about 18^+. Charge reduction afforded additional stability for the GIRK2 complex in the gas-phase, where more than half of the charge states' arrival-times fall on a trend-line that is typically associated with "native-like" protein complexes.

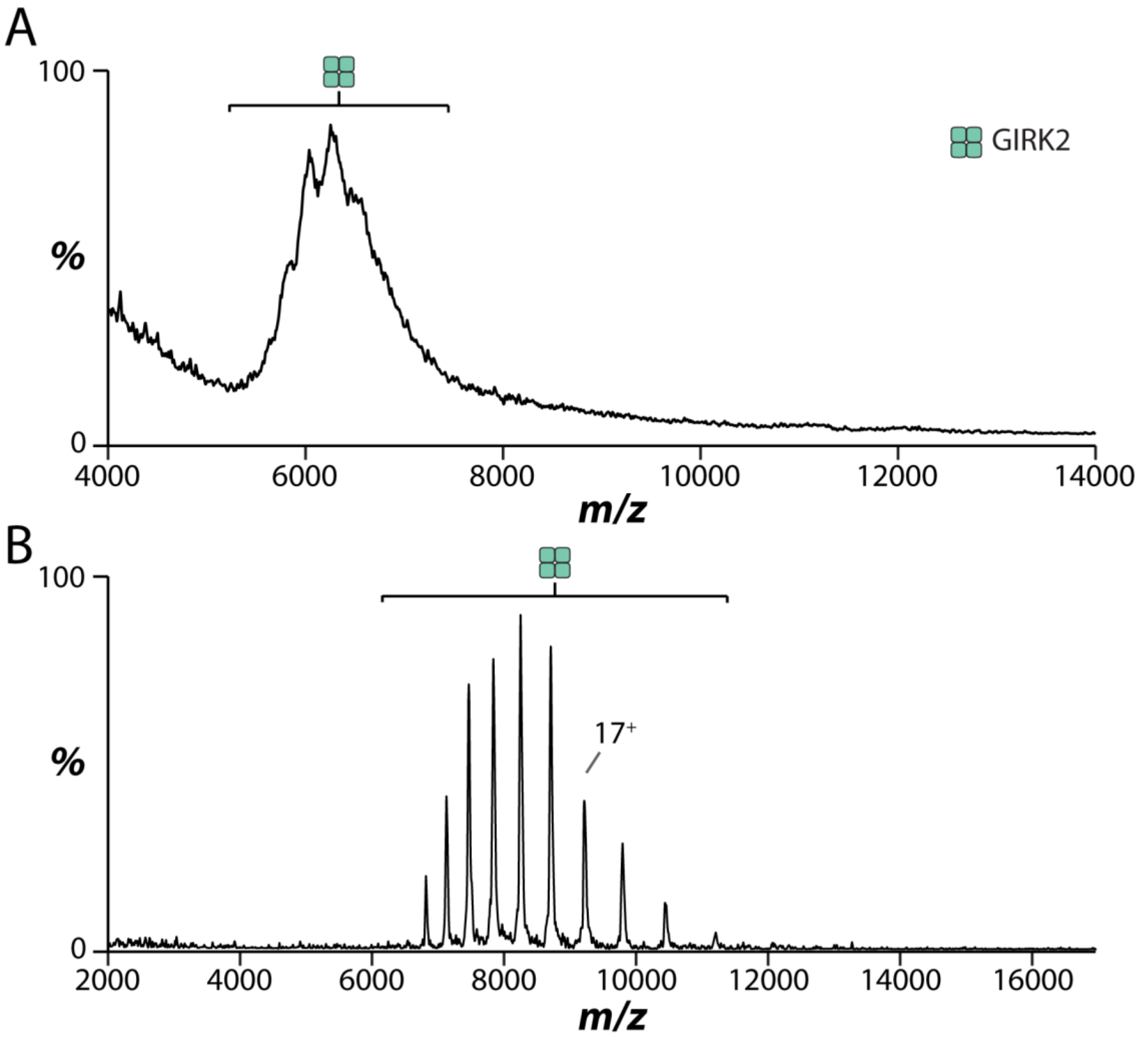

Supplementary Figure 3. Optimization of sample purification is required to obtain a resolved native mass spectrum. A) Native mass spectrum of GIRK2 purified following previously established methods[1] in DDM collected at the same instrument conditions as in Figure 1C yields a hump with a unresolved mass spectrum. B) Optimized sample in $C_{10}E_5$ after a DHPC treatment as described in methods. The activation energies were 120 V and 80 V for the trap and transfer cell, respectively. No dissociated monomers or stripped oligomers are observed under these experimental conditions for GIRK2 liberated from $C_{10}E_5$.

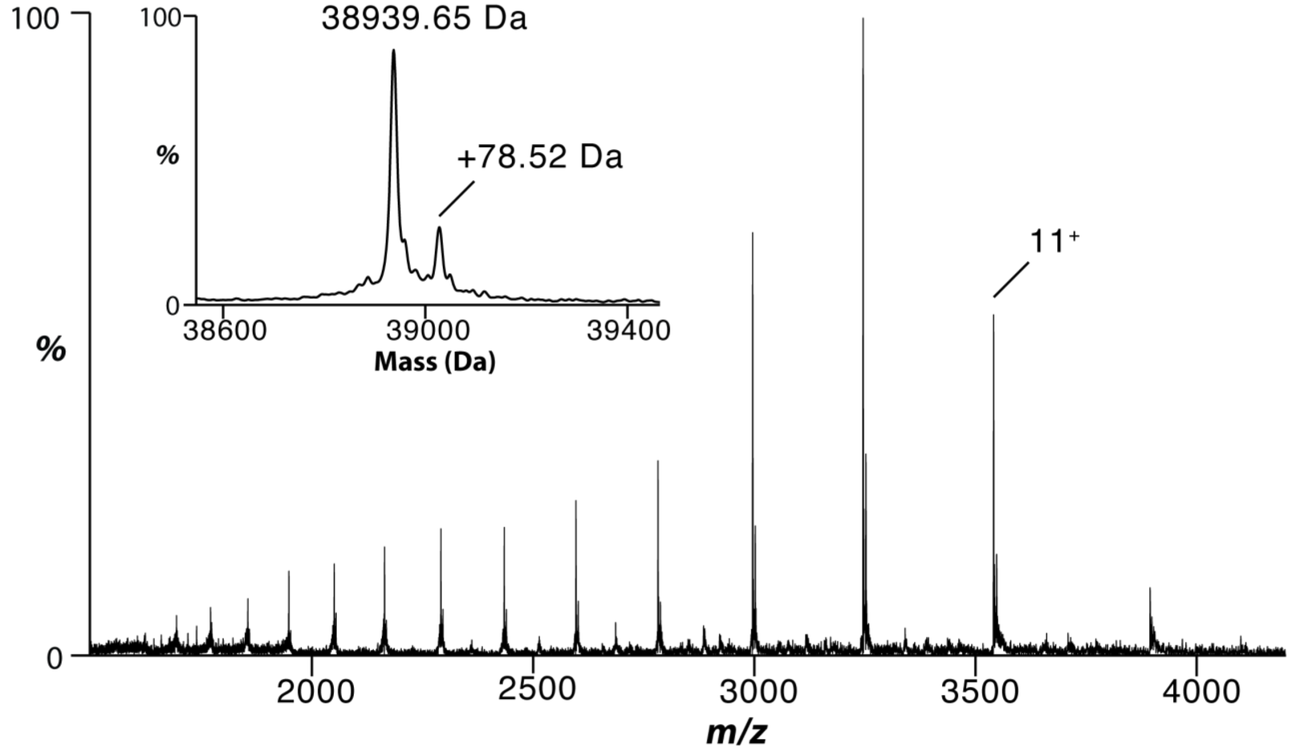

Species	Calculated (Da)	Observed (Da)	Δ Mass (Da)
GIRK2	38941.85*	38939.65 ± 0.82	2.20
Phosphorylation	78.97	78.52 ± 0.82	0.45

Supplementary Figure 4. Mass spectrum of intact and denatured GIRK2. Inset shows zero-charge mass spectrum with labeled mass species. The table shows the summary of the mass species. *Monomer mass was calculated with the initiating methionine removed. Roughly 19% of signal intensity is attributed to the phosphorylated species.

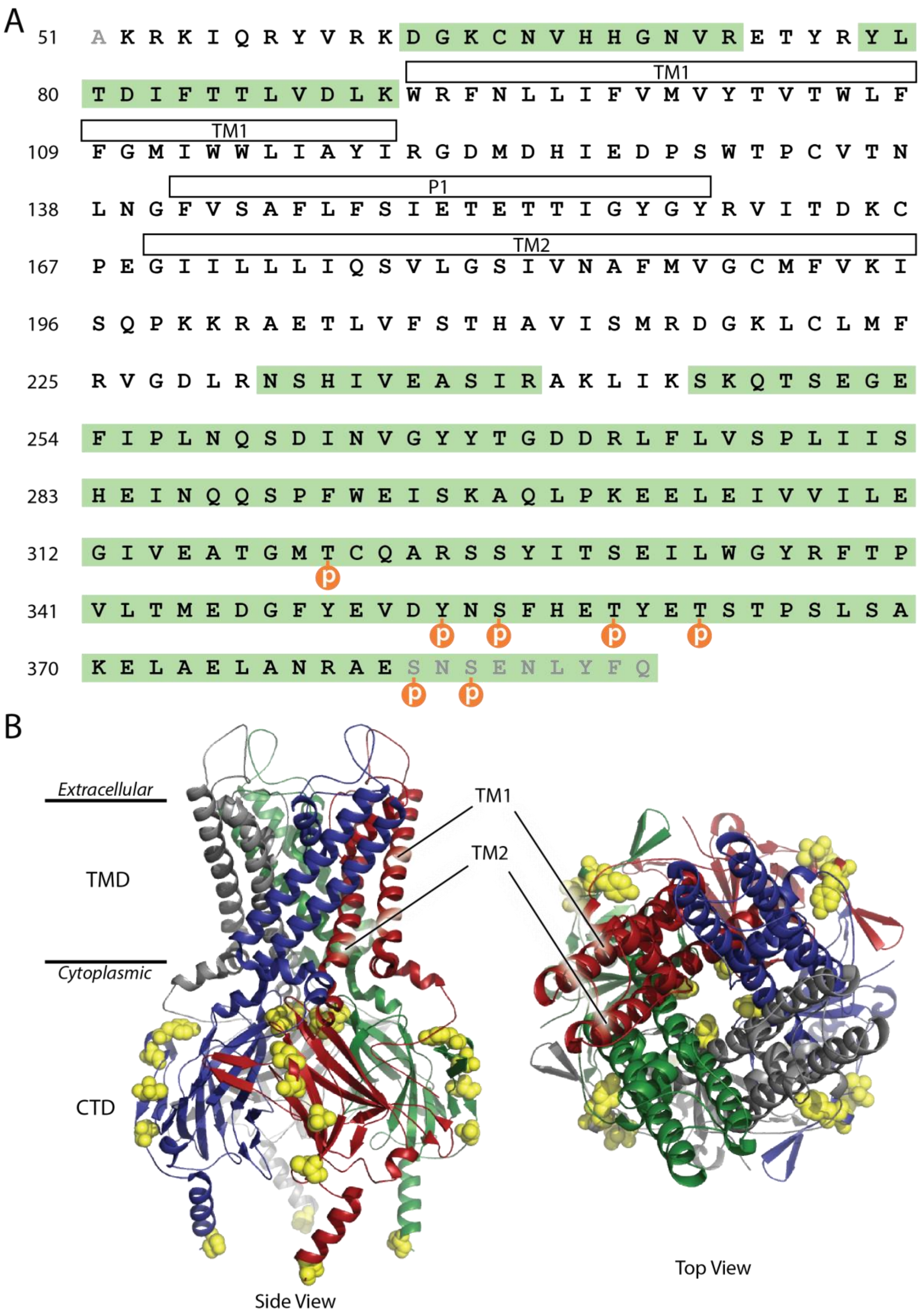

Supplementary Figure 5. Bottom-up MS analysis of GIRK2 tryptic peptides. A) Sequence coverage (53%) is shown in green and identified phosphorylation sites labeled with an orange circle. Non GIRK2 residues from the expression construct are shown in grey colored font. B) Structural of GIRK2 (PDB 3SYA) with identified phosphorylation sites shown as yellow spheres.

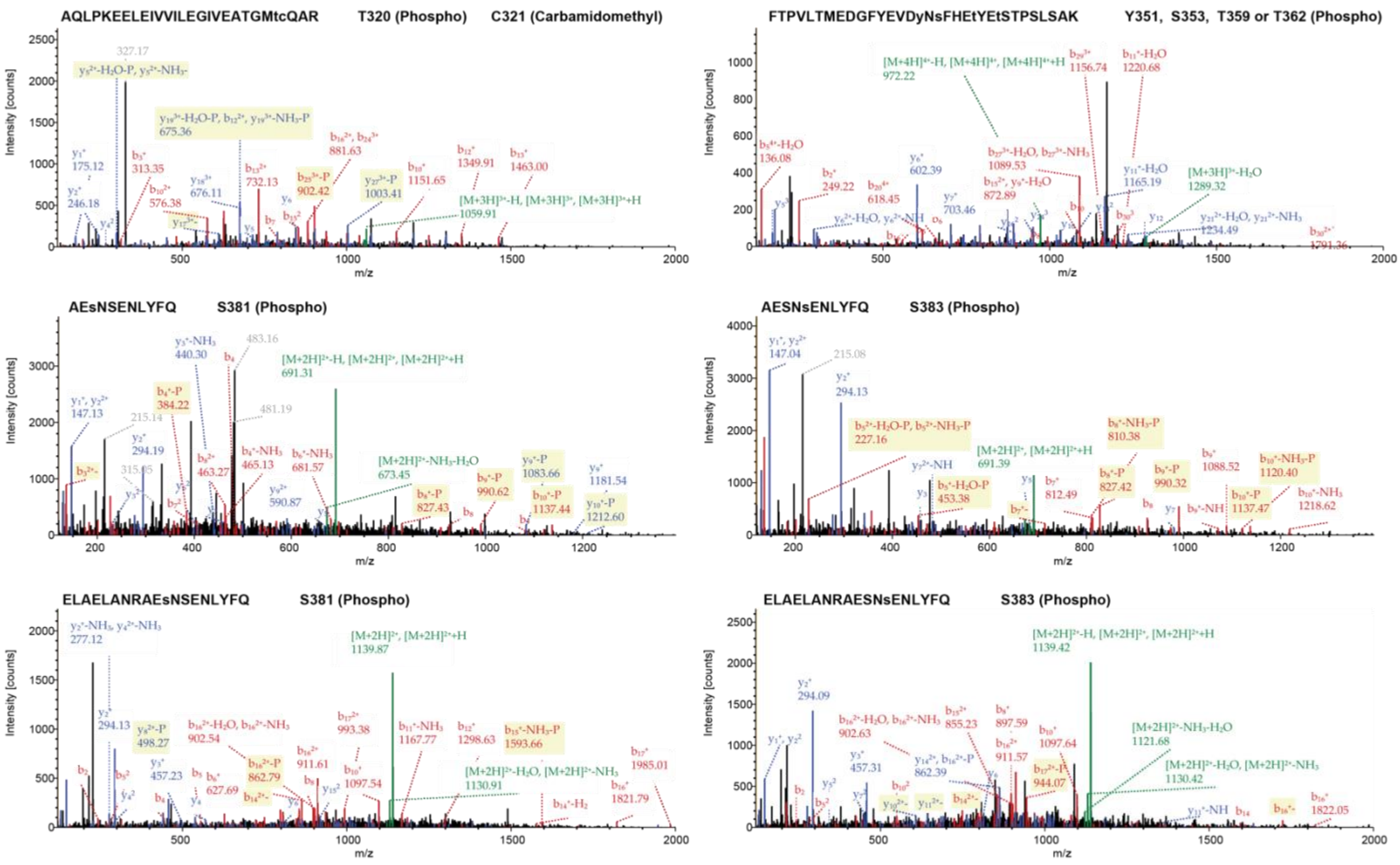

Supplementary Figure 6. Representative MS/MS spectra of singly phosphorylated tryptic peptides. B and y fragment ions are shown in red and blue, respectively. Peptide sequence and covalent modification site (lower case letters) is shown.

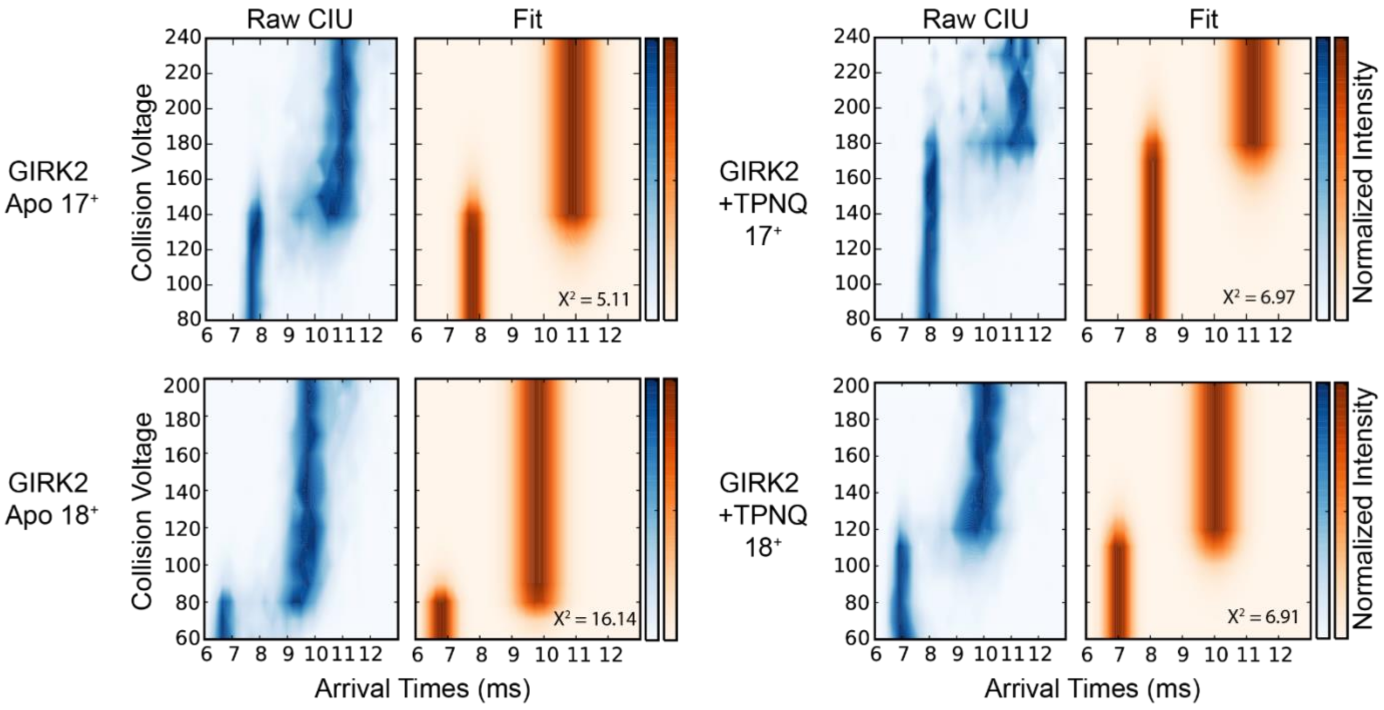

Supplementary Figure 7. CIU unfolding profiles for 17^+ and 18^+ charge states of apo and TPNQ bound GIRK2. The blue plots represent experimental data and orange plots are fits to the experimental data using the software program, PULSAR.[5] Reported in the fitted models is the chi-square fitting statistic from PULSAR. TPNQ binding to GIRK2 significantly stabilizes the channel as evident by large shift in the transition from native-like to a partially unfolded state.

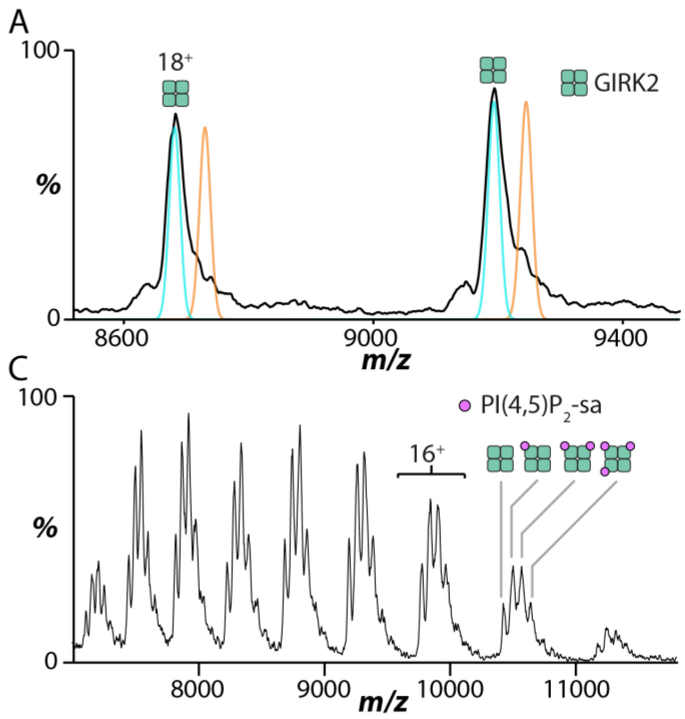

Supplementary Figure 8. Native mass spectra of GIRK2 doped with ivermectin and/or PI(4,5)P. (A) Representative mass spectrum of GIRK2 showing the 17^+ and 18^+ charge states. Blue lines show the estimated resolution achieved on the GIRK2 complex, and orange lines show hypothetical ivermectin binding events at the same resolving power. (B) Representative mass spectrum showing 17^+ and 18^+ charge state for GIRK2 in the presence of 300 µM ivermectin which is solubilized in MS buffer containing 5% ethanol and 3% DMSO. No binding of ivermectin was observed at the calculated *m/z* values for either wild-type or GIRK2^{R201A} (data not shown for GIRK2^{R201A} or concentrations below 300 µM) (C) Mass spectrum of mixture containing 500 nM GIRK2 and 3 µM PI(4,5)P_2-sa, the most abundance form of PI(4,5)P_2 in mammalian cells [10]. (D) Native mass spectrum for a mixture of 500 nM GIRK2, 3µM PI(4,5)P_2-sa and 50 µM ivermectin. No changes to the lipid binding pattern is observed in the presence of ivermectin, but there is a shift in the overall charge-state distribution.

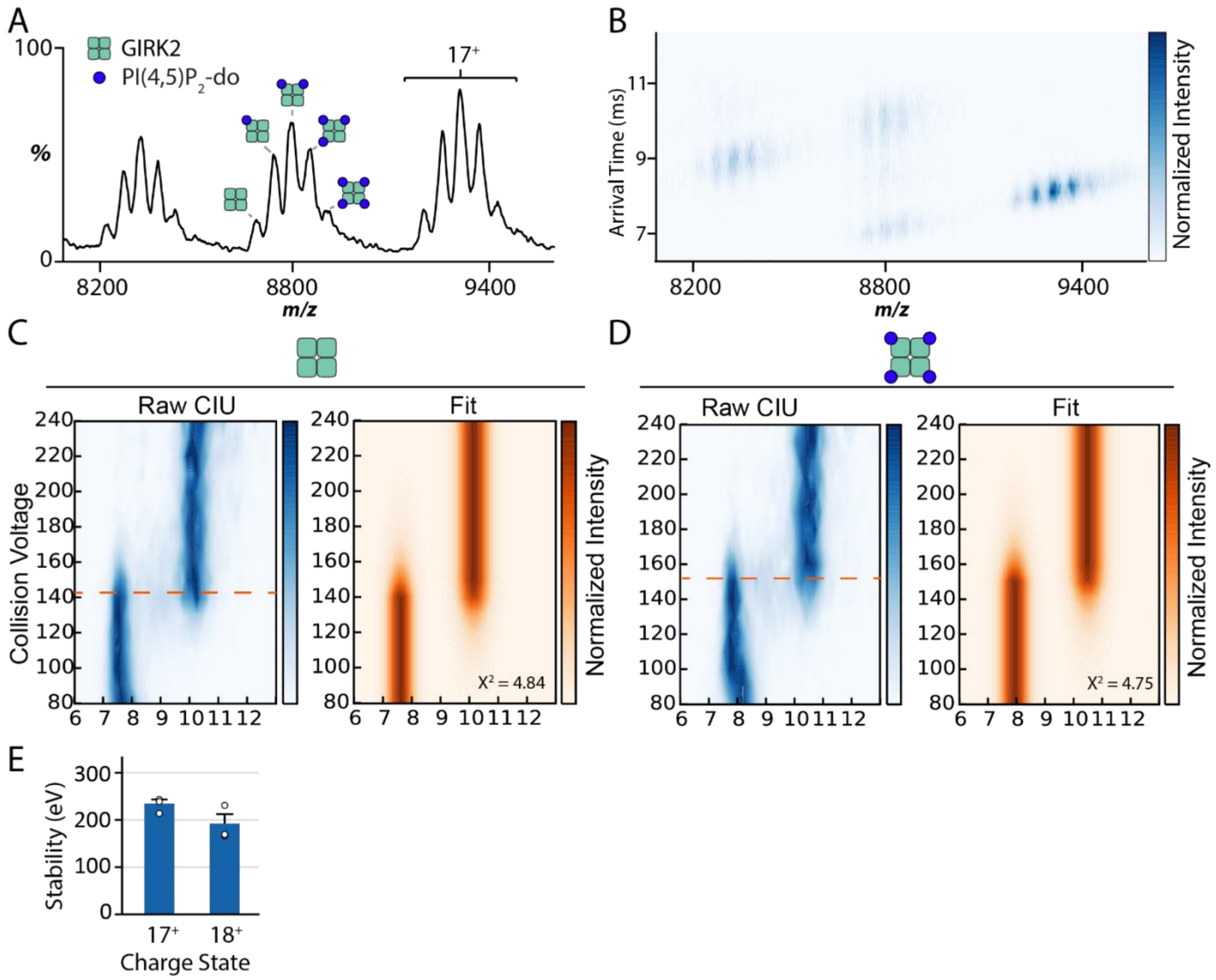

Supplementary Figure 9. Collision inducing unfolding plots for apo and PIP2 bound GIRK2. (A) and (B) shows the mass and ion-mobility spectrum for GIRK2 bound to PI(4,5)P2-do, respectively. (C) and (D) shows the CIU profiles and the fitted model for unfolding of apo GIRK2 and GIRK2 bound to four PI(4,5)P2-do molecules, respectively. (E) Stabilization calculated from parameters defined by fitting GIRK 17^{+} and 18^{+} bound to four PI(4,5)P2-do molecules. Reported are average and standard deviation from repeated measurements (*n*=3) in electron volts (eV).

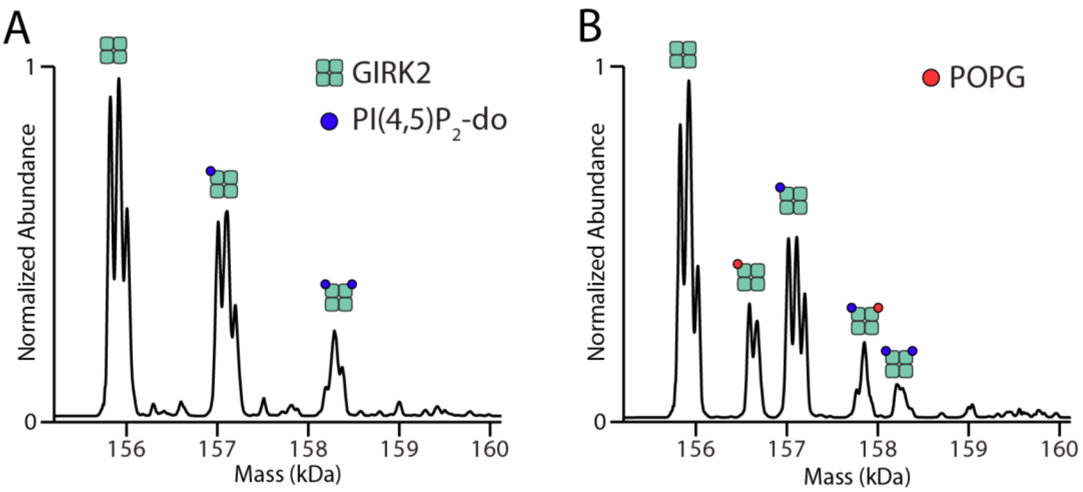

Supplementary Figure 10. Deconvolution of high-resolution native mass spectra of GIRK2 bound to different lipids. (A) GIRK2 incubated with four molar equivalents of PI(4,5)P_2-do. (B) GIRK2 incubated with a equimolar mixture of POPG and PI(4,5)P_2-do. For the mixture, no change in binding of PI(4,5)P_2-do is observed that would indicate competition or positive allosteric modulation.

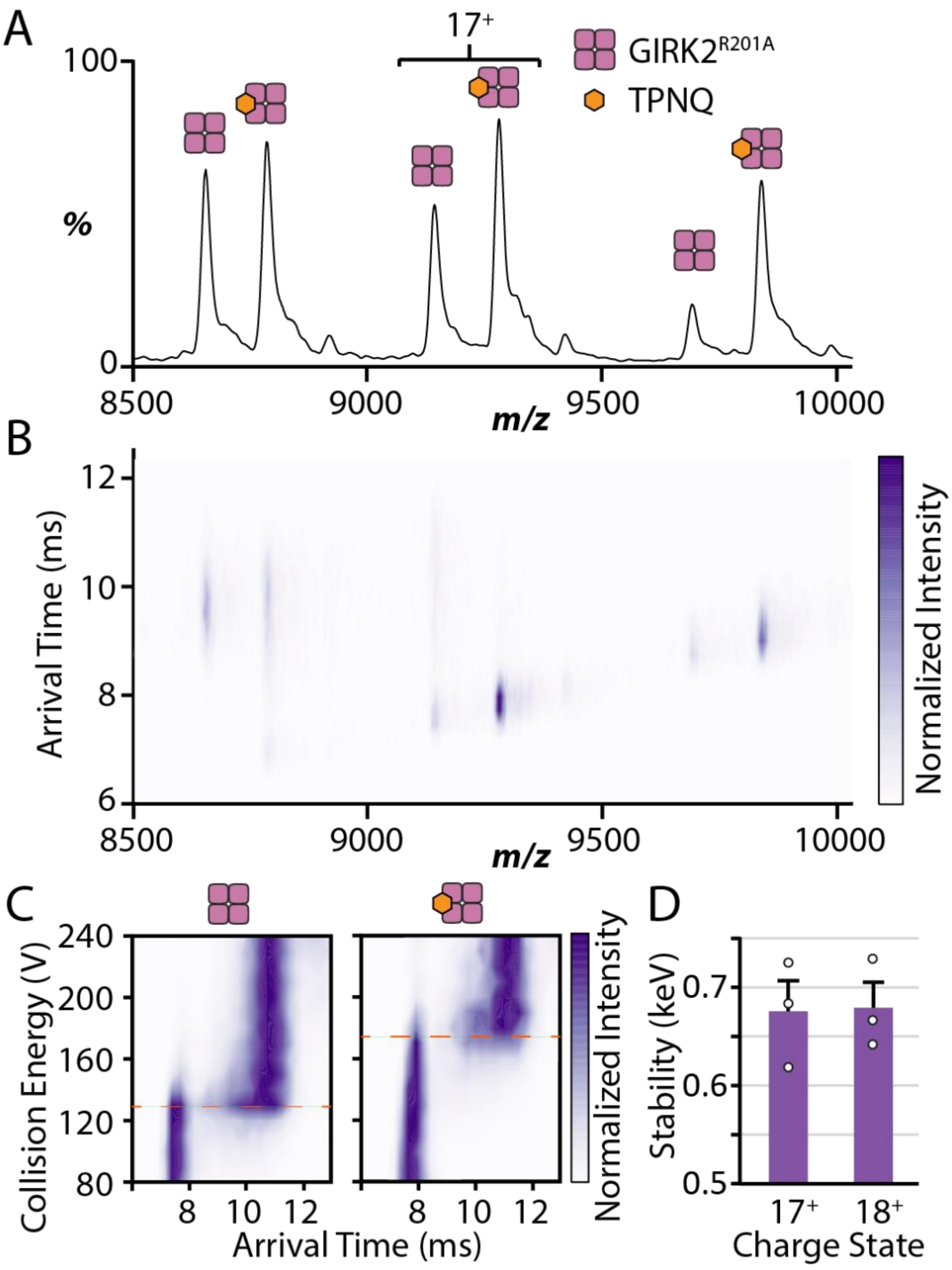

Supplementary Figure 11. GIRK2^{R201A} mixed with TPNQ reveals the bound toxin significantly stabilizes the channel. (A) Native mass spectrum of GIRK2 mixed with 5 µM TPNQ. (B) Ion mobility mass spectrum showing the arrival times for 16^+, 17^+ and 18^+ charge states. (C) CIU profiles for the 17^+ charge state apo and TPNQ bound GIRK2^{R201A}, orange dotted line indicates the calculated transition voltage from PULSAR.[5] (D) Stabilization calculated from parameters defined by fitting GIRK2 (17^+ and 18^+) bound to TPNQ. Reported are average and standard deviation from repeated measurements (*n*=3) in electron volts (eV). Stabilization of the mutant channel by TPNQ is statistically similar to wild-type channel.

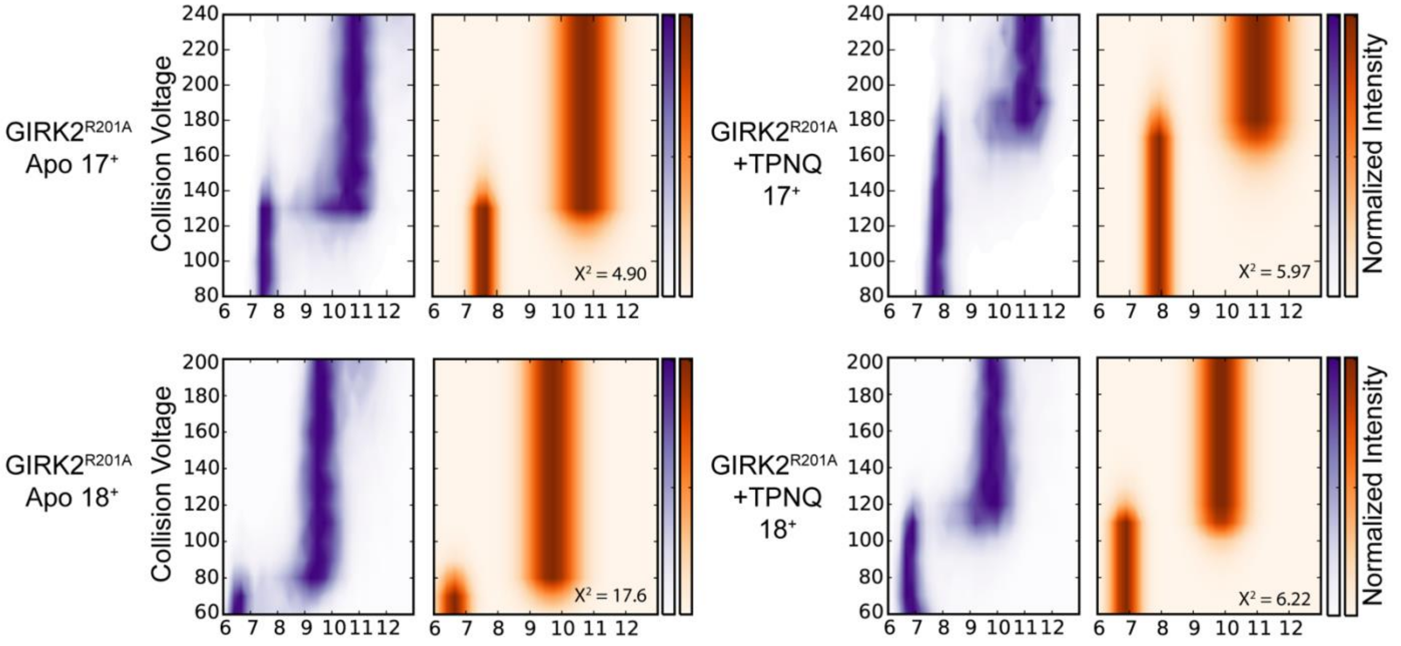

Supplementary Figure 12. CIU unfolding profiles for both 17^+ and 18^+ charge states of apo and TPNQ bound $GIRK2^{R201A}$. Shown as described in Supplementary Figure 4.

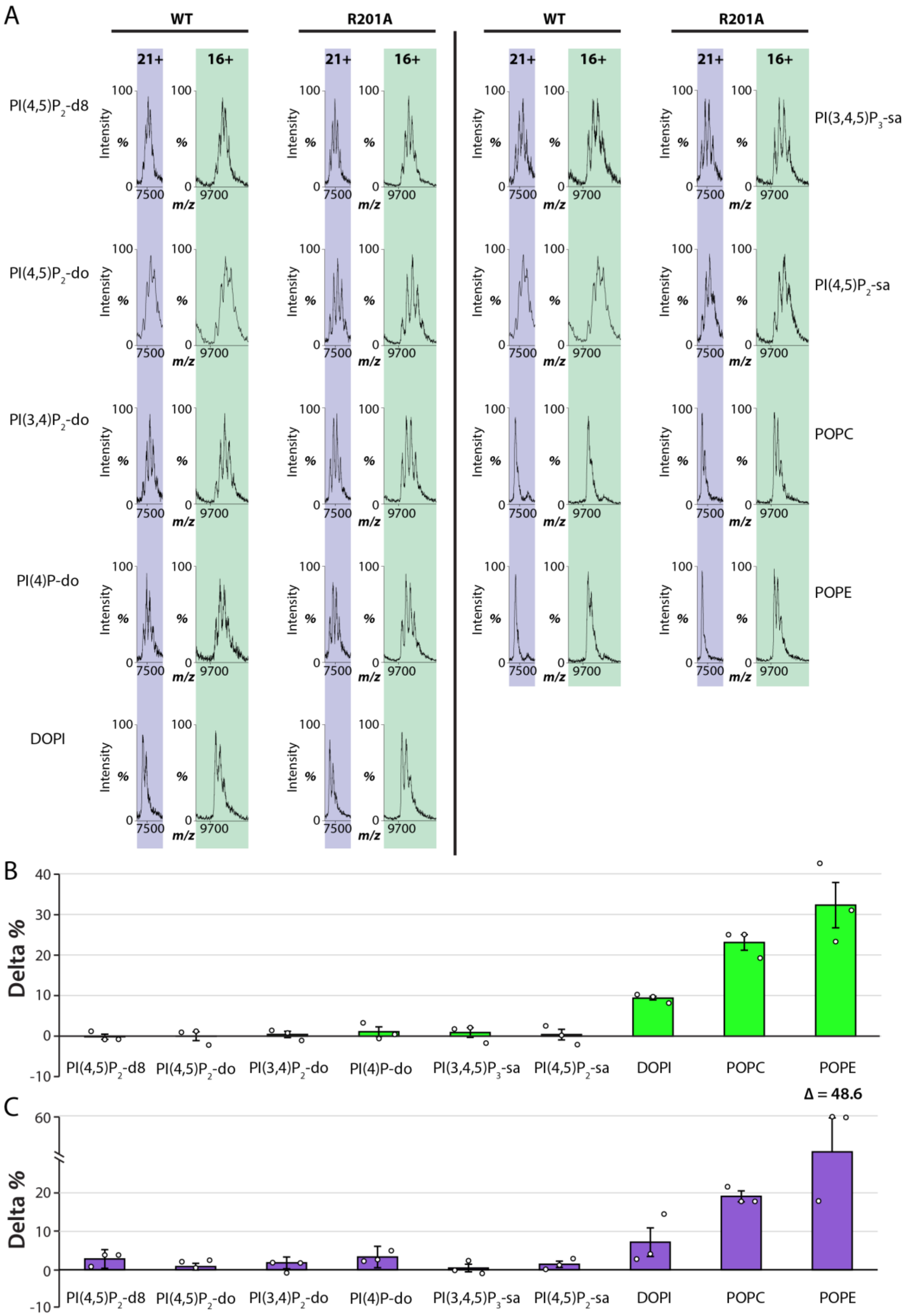

Supplementary Figure 13. Comparison between partially activating (21^+) and native-like (16^+) charge states for different lipids bound to GIRK2. (A) Enlargement of regions of mass spectra for 21^+ and 16^+ charge states of GIRK2 and GIRK2^{R201A} bound to different lipids. For all PIPs, the fractional abundances of apo and lipid-bound GIRK2 are very similar between the two charge states. However, the other lipids (PI, PC and PE) display a dramatic reduction in the amount of lipid retained at the higher charge state. Calculated differences between the mole fraction of lipids bound to the 16^+ compared to the 21^+ charge state of (B) wild-type and (C) R201A mutant GIRK2.

Supplementary References

1 Whorton, M. R. & MacKinnon, R. Crystal structure of the mammalian GIRK2 K+ channel and gating regulation by G proteins, PIP2, and sodium. *Cell* **147**, 199-208, doi:10.1016/j.cell.2011.07.046 (2011).

2 Gandhi, C. S., Walton, T. A. & Rees, D. C. OCAM: a new tool for studying the oligomeric diversity of MscL channels. *Protein Sci* **20**, 313-326, doi:10.1002/pro.562 (2011).

3 Cong, X. *et al.* Determining Membrane Protein-Lipid Binding Thermodynamics Using Native Mass Spectrometry. *J Am Chem Soc* **138**, 4346-4349, doi:10.1021/jacs.6b01771 (2016).

4 Laganowsky, A. *et al.* Membrane proteins bind lipids selectively to modulate their structure and function. *Nature* **510**, 172-175, doi:10.1038/nature13419 (2014).

5 Allison, T. M. *et al.* Quantifying the stabilizing effects of protein-ligand interactions in the gas phase. *Nat Commun* **6**, 8551, doi:10.1038/ncomms9551 (2015).

6 Marty, M. T. *et al.* Bayesian deconvolution of mass and ion mobility spectra: from binary interactions to polydisperse ensembles. *Anal Chem* **87**, 4370-4376, doi:10.1021/acs.analchem.5b00140 (2015).

7 Poltash, M. L., McCabe, J. W., Patrick, J. W., Laganowsky, A. & Russell, D. H. Development and Evaluation of a Reverse-Entry Ion Source Orbitrap Mass Spectrometer. *J Am Soc Mass Spectrom*, doi:10.1007/s13361-018-1976-0 (2018).

8 Lippens, J. L. *et al.* Rapid LC-MS Method for Accurate Molecular Weight Determination of Membrane and Hydrophobic Proteins. *Anal Chem* **90**, 13616-13623, doi:10.1021/acs.analchem.8b03843 (2018).

9 Whorton, M. R. & MacKinnon, R. X-ray structure of the mammalian GIRK2-betagamma G-protein complex. *Nature* **498**, 190-197, doi:10.1038/nature12241 (2013).

10 Traynor-Kaplan, A. *et al.* Fatty-acyl chain profiles of cellular phosphoinositides. *Biochim Biophys Acta* **1862**, 513-522, doi:10.1016/j.bbalip.2017.02.002 (2017).
